# Influenza A virus hemagglutinin glycosylation compensates for antibody escape fitness costs

**DOI:** 10.1371/journal.ppat.1006796

**Published:** 2018-01-18

**Authors:** Ivan Kosik, William L. Ince, Lauren E. Gentles, Andrew J. Oler, Martina Kosikova, Matthew Angel, Javier G. Magadán, Hang Xie, Christopher B. Brooke, Jonathan W. Yewdell

**Affiliations:** 1 Cellular Biology Section, Laboratory of Viral Diseases, NIAID, Bethesda, Maryland, United States of America; 2 Center for Drug Evaluation and Research, FDA, Silver Spring, Maryland, United States of America; 3 Bioinformatics and Computational Biosciences Branch, Office of Cyber Infrastructure and Computational Biology, NIAID, Bethesda, Maryland, United States of America; 4 Laboratory of Respiratory Viral Diseases, Division of Viral Products, Office of Vaccines Research and Review, Center for Biologics Evaluation and Research, FDA, Silver Spring, Maryland, United States of America; 5 Department of Microbiology, School of Molecular and Cellular Biology, University of Illinois, Urbana, Illinois, United States of America; 6 Carl R. Woese Institute for Genomic Biology, University of Illinois, Urbana, Illinois, United States of America; St. Jude Children’s Research Hospital, UNITED STATES

## Abstract

Rapid antigenic evolution enables the persistence of seasonal influenza A and B viruses in human populations despite widespread herd immunity. Understanding viral mechanisms that enable antigenic evolution is critical for designing durable vaccines and therapeutics. Here, we utilize the primerID method of error-correcting viral population sequencing to reveal an unexpected role for hemagglutinin (HA) glycosylation in compensating for fitness defects resulting from escape from anti-HA neutralizing antibodies. Antibody-free propagation following antigenic escape rapidly selected viruses with mutations that modulated receptor binding avidity through the addition of N-linked glycans to the HA globular domain. These findings expand our understanding of the viral mechanisms that maintain fitness during antigenic evolution to include glycan addition, and highlight the immense power of high-definition virus population sequencing to reveal novel viral adaptive mechanisms.

## Author summary

Seasonal influenza A viruses (IAV) cause tens of thousands of deaths and tens of billions of dollars in economic costs every year in the U.S. alone, despite widespread pre-exposure and vaccination. IAV persists within the human population by evading herd immunity through the continual accumulation of immune escape substitutions in a process known as antigenic drift. Unfortunately, the specific mechanisms that facilitate IAV antigenic drift remain unclear, making it difficult to design more efficacious, escape-proof vaccines. Here, we used a high-definition population sequencing approach to understand how IAV tolerates the accumulation of immune escape substitutions during antigenic drift without losing fitness. We found that the virus rapidly acquired N-linked glycan structures on the hemagglutinin (HA) protein following escape from a panel of neutralizing antibodies. Glycosylation of HA alleviated the deleterious effects of immune escape substitutions on interactions with host cell receptors. These results expand our understanding of the mechanisms that facilitate IAV immune evasion, and highlight the immense power of high-definition virus population sequencing to reveal novel viral adaptive mechanisms.

## Introduction

Influenza A virus (IAV) persists in human populations by continuously evolving to escape herd immunity. Protective immunity is predominantly mediated by neutralizing antibodies (Abs) specific for the viral surface glycoprotein hemagglutinin (HA). HA mediates both target cell attachment, by binding terminal sialic acids (SA) on cellular membrane components, and fusion between viral and cellular membranes following virion internalization. Neutralizing Abs mainly target the five highly variable immunodominant antigenic sites on the globular head domain of HA, blocking either HA-mediated attachment or fusion [1–4]. Viruses can escape neutralization by amino substitutions in HA that reduce antibody affinity and/or function. The fitness costs imposed by Ab escape, and the mechanisms by which the virus compensates remain poorly understood, yet play a central role in governing HA antigenic evolution [5,6].

Influenza virus escapes from neutralizing antibody via a number of defined mechanisms involving HA mutations. The simplest is an amino acid substitution in a cognate epitope that diminishes antibody affinity [2]. Less commonly, distant substitutions can affect antibody binding via allosteric effects on antibody access to its epitope [7–9]. Amino acid substitutions in the sialic acid receptor site, or other regions of the globular domain that increase affinity for cellular SA receptors, enable Ab-escape by shifting the binding equilibrium towards virus binding to cells *versus* antibody [10–13]. The most drastic alterations in overall antigenicity typically result from amino acid substitutions that create a N-linked glycosylation site in the globular domain, as the attached glycan can sterically block the binding of Abs to multiple sites [5,14–19].

The ability of viruses to exploit these escape routes is limited by their costs to viral fitness. The sialic acid receptor binding site is nestled among the immunodominant antigenic sites, and escape substitutions often alter viral fitness by changing HA receptor binding avidity. Amino acid substitutions that change the net charge of the globular domain typically alter cell binding avidity [13,20]. We previously demonstrated that selection of substitutions that add N-linked glycosylation sites within the globular domain to escape antibody neutralization often reduces receptor avidity [5]. Receptor avidity and HA antigenicity are thus intimately linked, and must be continuously balanced to maintain fitness during antigenic evolution [13,21].

Here, we use the primerID method for error-correcting virus population sequencing to reveal a surprising new role for N-linked glycosylation in facilitating IAV immune escape. Beyond its canonical role in blocking Ab binding, we show that glycosylation can also compensate for fitness costs imposed by escape substitutions elsewhere on HA, thus increasing the viability and subsequent emergence potential of Ab-escape variants.

## Results

### HA Ab escape mutations reduce viral fitness in the absence of Ab pressure

We previously modeled IAV antigenic evolution by sequentially selecting for virus escape from different mouse anti-HA monoclonal Abs (mAbs) under over-neutralizing conditions using the allantois-on-shell (AOS) system [21]. Twelve rounds of mAb escape by PR8 yielded a virus (SV12) carrying twelve amino acid substitutions in HA that collectively mediated near total escape from polyclonal serum raised against PR8 in mice, guinea pigs or chickens. When propagated in embryonated chicken eggs, PR8 and SV12 reached similar titers in the absence of Ab pressure, suggesting that the selected constellation of escape mutations imposed minimal fitness costs on the virus.

**Fig 1:**
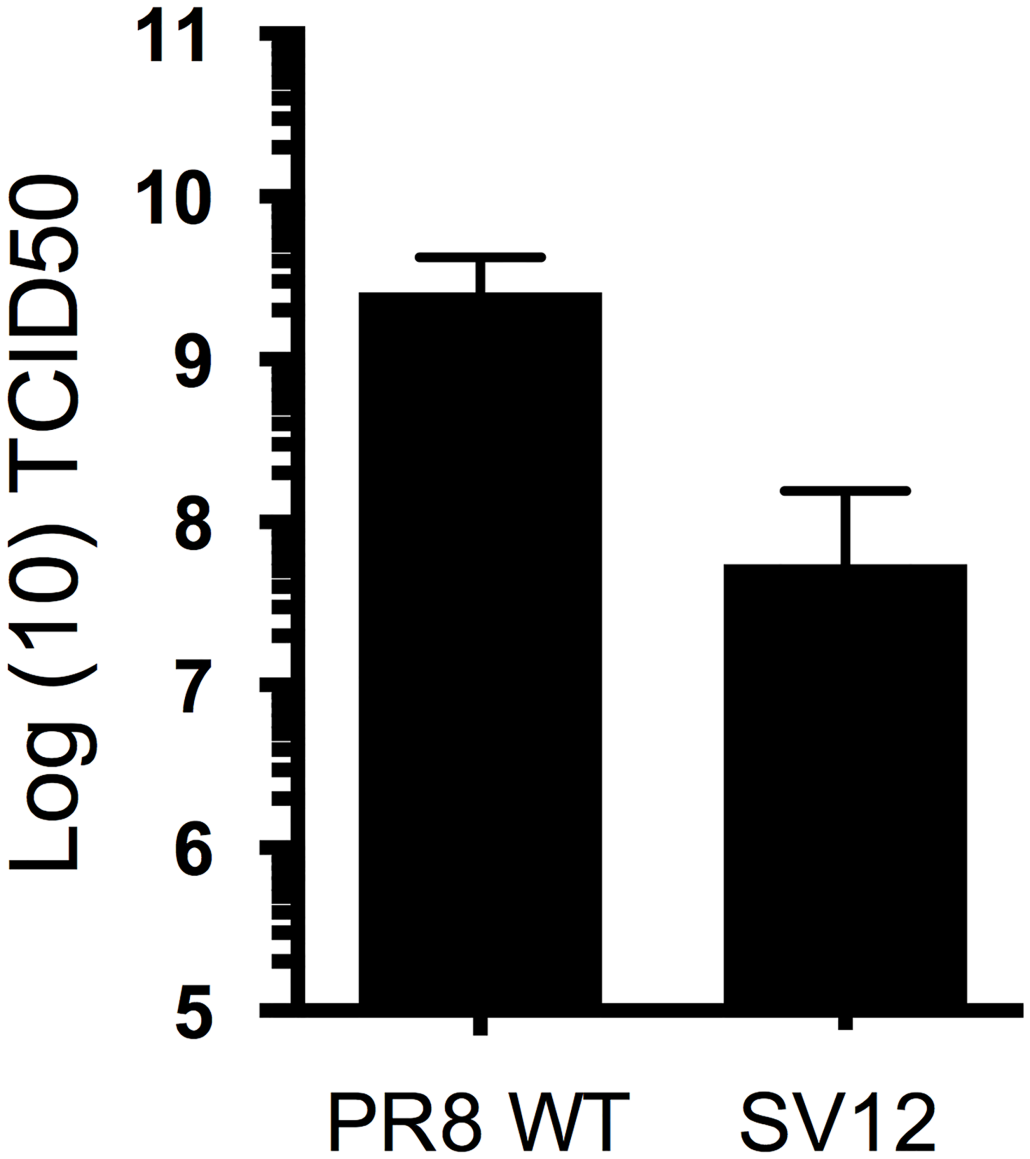
Fitness tradeoff associated with the accumulation of antigenic escape substitutions in HA. Growth of plaque purified biological clones of PR8 WT and SV12 was compared in eggs. Eggs were infected in triplicate with 500 TCID50 of the indicated virus. 48 hours later, viral loads in allantoic cavities were titered by TCID50 assay.

An alternative possibility that we did not examine in detail is that the accumulation of antigenic escape substitutions imposed significant costs, but strong selection for compensatory mutations or reversions in SV12 rapidly restored fitness during the initial rounds of SV12 expansion in eggs; the original SV12 isolate had been expanded 2–3 times in the absence of antibody in embryonated chicken eggs after the 12^th^ sequential selection in the AOS system [21]. To test this possibility, we generated a recombinant PR8 clone carrying the consensus SV12 HA gene segment and compared the ability of WT PR8 and the consensus SV12 clone to replicate in eggs (Fig 1). This revealed that antigenic escape substitutions present within SV12 HA imposed significant fitness costs (greater than 10-fold decreased titer) when the virus population had limited time to recover fitness via mutational compensation.

### High resolution examination of IAV mutational spectra using primerID sequencing

Compensatory mutations that increased the fitness of SV12 may not have been identified with conventional Sanger sequencing if multiple variants were selected that collectively were a significant proportion of the population, but that individually remained below the wild-type SV12 consensus frequency at each codon. To identify compensatory mutations or reversions in HA that potentially emerged following sequential antigenic escape, we used the primerID method for error-correcting virus population sequencing to identify minor sequence variants that emerged during the limited expansion passaging of SV12 [22,23].

PrimerID sequencing is based on tagging individual viral cDNAs with random unique sequence barcodes during reverse transcription [22,23]. These barcodes are maintained through downstream PCR and sequencing steps and are used to assemble all sequencing reads derived from a single cDNA sequence. This permits consensus-based reconstruction of the original cDNA sequence, enumeration of actual viral genomes sampled, and analysis of linkage between specific mutations within reads. Importantly, primerID lowers the background sequencing error rate of the Illumina platform 10-100-fold (to 10^−3^–10^−4^ non-consensus events/nt), by correcting the high-frequency PCR and base-calling errors that occur during standard next-generation sequencing. PrimerID can also correct for variant frequency skewing due to PCR resampling imbalance [22,23].

To precisely quantify the advantages conferred by primerID sequencing over shotgun sequencing approaches in the context of IAV population sequencing, we directly compared results obtained with the primerID method to those obtained by shotgun sequencing, as analyzed through the ViVan pipeline [24]. Variant calls with a moderately high frequency (>10^−3^) show consistent results whether an amplicon/primerID or a shotgun sequencing approach were used (S1 Fig); however, evaluating variant frequencies in the range of 10^−4^ to 10^−3^ shows that primerID gives a higher signal to background noise ratio compared to shotgun sequencing (S2 Fig). The noise reduction of primerID consensus reads compared to unmerged amplicon reads ranges from 9.2-fold to 18.9-fold (S1 Table). Similarly, the background noise level, estimated by the median combined minor allele frequency (MAF) in primerID is about 3-10x10^-5^, which is 4.5 to 12-fold lower than background in shotgun sequencing (S2 Table). Altogether, primerID allows for more conservative variant calling, and likely more accurate estimations of variant frequencies, than algorithm-based error correction approaches [24,25].

### PrimerID population sequencing reveals the rapid emergence of compensatory variants following antigenic escape

**Fig 2:**
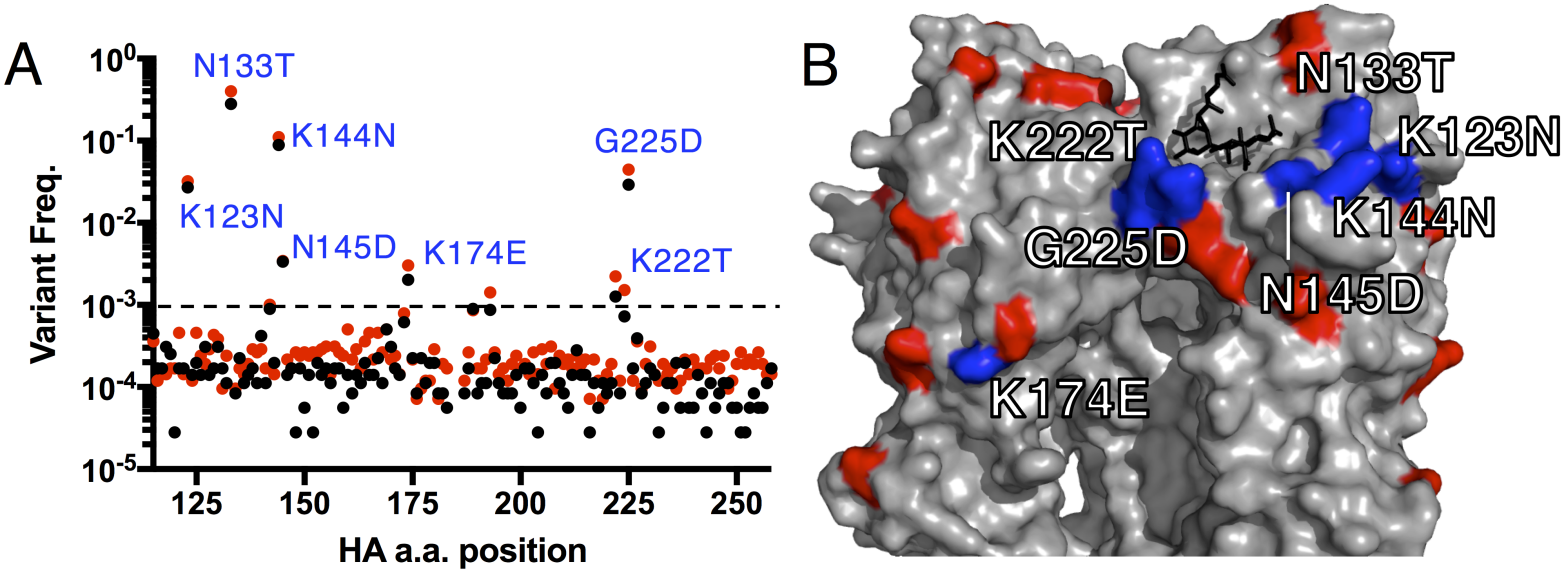
Identification of variants that emerge on the SV12 background after passage in eggs in the absence of antibody. **(A)** Amino acid variant frequencies within SV12 HA following limited passage in eggs, as determined by primerID sequencing. Red and black data points represent frequencies obtained from two stocks separately expanded one time in eggs prior to sequencing. Amino acid positions with >0.1% variability (dashed line) in both stocks are labeled with the most abundant substitution at the position. Codons 184–186 were not captured by sequencing. **(B)** HA structure (PDB 1RU7) with the SV12-defining substitutions (red) and emerging amino acid substitutions (labeled in 2A) (blue) highlighted. The black stick model represents sialic acid situated in the receptor binding pocket.

To identify emergent HA variants within an approximately 143 a.a. region (115 to 258) that encompassed the receptor binding pocket and most of the SV12 substitutions in the original expanded SV12 virus stock, we performed paired-end sequencing of primerID libraries on the Illumina MiSeq platform and analyzed data using a custom software pipeline. Sequencing revealed amino acid substitutions at 7 sites that emerged at frequencies of >0.1% during 2–3 passages of SV12. These substitutions were (including their frequencies) K123N (1.7%), K123E (1.1%), N133T (25%), N133S (8%), K144N (9%), K144E (0.5%), N145D (0.3%), K174E (0.3%), K222T (0.17%), and G225D (3.7%) (all H3 HA numbering) (Fig 2A). Linkage analysis revealed that substitutions at positions K123, N133, K144, K145, and G225 represented distinct variants (S2 Table), and thus collectively made up approximately 50% of the population of the SV12 stock.

Each substitution was observed at sites with potential functional roles in SV12 (Fig 2B). Substitutions at two SV12-defining sites were observed: G225D was a reversion to the PR8 wild-type amino acid identity, while the most abundant substitution at N145 was D (0.3%), rather than the PR8 wild-type S (0.01%). The other substitutions (K123N/E, N133T/S, K144N/E, K174E, and K222T) were at new sites, but were adjacent to SV12-defining sites or rimmed the receptor binding site (Fig 2B). Thus, a combination of forward mutations and reversions rapidly emerged on the SV12 background following antigenic escape.

### The addition of N-linked glycans to HA can compensate for the fitness costs of antigenic escape

**Fig 3:**
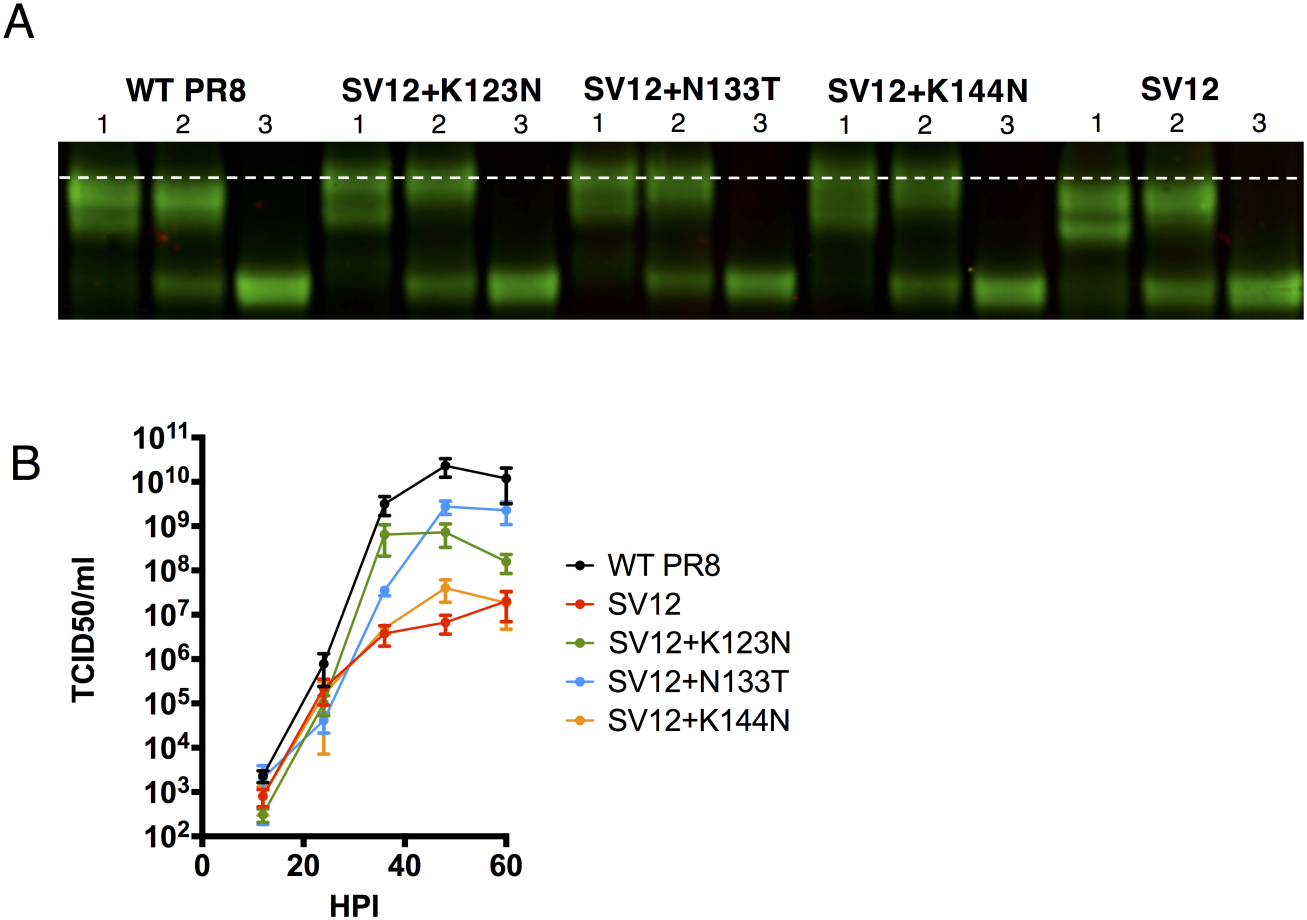
Characterization of compensatory glycan additions. **(A)** Western blot comparison of HA glycosylation mutants following endoglycosidase treatment. Treatment 1 = undigested, treatment 2 = Endo H digested, treatment 3 = PNGase digested. **(B)** Growth comparison of PR8, SV12, and SV12 plus individual compensatory mutations in eggs. Eggs were inoculated with 500 TCID50, and viral loads within allantoic cavities were assessed by TCID50 assay at the indicated time points.

Three of the amino acid substitutions that emerged in the original expanded SV12 virus stock (K123N, N133T, and K144N) created new potential N-linked glycan sites (PNGS) within the HA sequence (at positions 123, 131 and 144, respectively). To determine whether these substitutions indeed affected the glycosylation status of virion-associated HA[26], we introduced each substitution individually into the recombinant consensus SV12 HA background (rSV12-HA) and rescued viruses carrying the 7 other gene segments from WT PR8 by reverse genetics. We generated purified, detergent-disrupted virus preparations of rSV12-HA, rSV12(K123N)-HA, rSV12(N133T)-HA, and rSV12(K144N)-HA and digested with either Endo H (cleaves mannose-rich N-linked glycans, but not complex N-linked glycans) or PNGase F (cleaves all N-linked glycans). We compared the migration of digested and undigested HA on a denaturing gel by immunoblotting (Fig 3A). All three compensatory PNGS clearly slowed the migration of undigested, but not PNGase F-digested, HA on the gel, demonstrating that each mutation results in the addition of a glycan chain to virion-associated HA.

We next tested whether compensatory glycosylation site additions increased the replicative capacity of rSV12-HA in multi-step growth assays in eggs (Fig 3B). The impact of K144N on titer was marginal, but K123N and N133T both resulted in 10–100 fold higher viral titers compared with parental rSV12-HA virus by 36 hpi, demonstrating a clear role for each of these glycosylation site additions in restoring fitness in eggs following SV12 antigenic escape.

### Glycan addition partially restores wild-type receptor binding characteristics following antigenic escape

**Fig 4:**
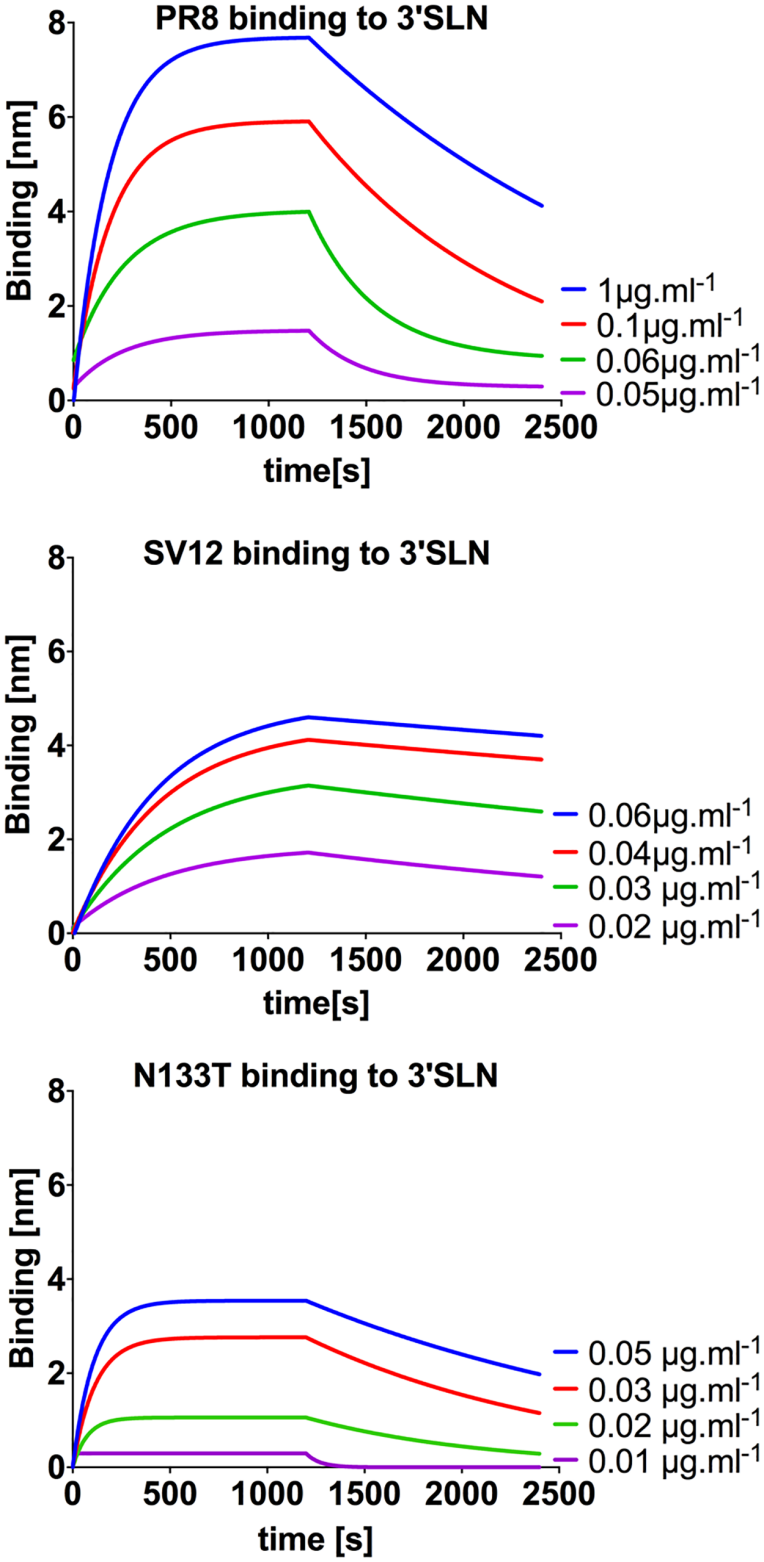
Bio-layer interferometry comparison of binding properties of WT, SV12 and SV12-N133T. To measure binding properties of WT PR8, SV12, and the compensatory glycan addition mutant N133T, we bound biotinylated 3’SLN to sensor at different concentrations, and measured the K_on_ and K_off_ of intact purified virions in the presence and absence of the NA inhibitor oseltamivir, respectively. We fit two-phase (association then dissociation) nonlinear regression curves based on the average of 2–3 experiments.

A clue to how these glycan additions restore SV12 fitness came from their close proximity to the HA receptor binding pocket (Fig 2B). We hypothesized that the glycans modulate HA receptor binding avidity, correcting for detrimental effects of escape substitutions. To test this, we compared the association (*K*_on_) and dissociation (*K*_*off*_) rates of purified WT PR8, rSV12-HA, rSV12(K123N)-HA, rSV12(N133T)-HA, and rSV12(K144N)-HA virion preps binding to the model sialic acid receptor 3-SLN using bio-layer interferometry (BLI) (Fig 4, Table 1). rSV12-HA exhibited a 3-fold increase in binding avidity, compared with WT HA. The addition of the K123N, and N133T substitutions to SV12 HA altered binding constants toward WT levels, consistent with our hypothesis. The K144N substitution reduced binding avidity to a much greater degree than K123N or N133T, resulting a 2-fold decrease in avidity relative to WT HA. This effect may have crossed a line beyond the beneficial effects of K123N and N133T, resulting in a detrimental loss of binding avidity (compared with WT) that may explain the comparatively slower growth kinetics of the K144N variant (Fig 3B).

**Table 1:** Bio-layer interferometry analysis of HA compensatory glycans.

	3’SLN K_on_ [nM^-1^.ms^-1^]	3’SLN K_off_ [μs^-1^]	3’SLN Apparent K_D_ [pM]	3’SLN R^2^
**PR8**	8.36±0.6	8.56±3.55	1.02±0.58	0.98
**K123N**	**14.38±1.36**	**8.34±1.36**	**0.44±0.24**	**0.99**
**N133T**	13.53±1.14	10.62±2.52	0.8±0.25	0.98
**K144N**	**4.56±0.02**	**9.59±2.18**	**2.1±0.49**	**0.95**
**N133T/K144N**	16.38±0.54	13.95±1.91	0.85±0.13	0.96
**SV12**	3.78±0.27	1.22±0.61	0.32±0.22	0.94

### Similar repertoires of compensatory mutations emerge during independent experiments

To determine the reproducibility (and potentially the predictability) of the compensatory mechanisms that we observed, we repeated the passage experiment using recombinant, reverse genetics-derived versions of either WT PR8 or SV12-HA (rSV12-HA) virus as the parental virus. We generated each virus in 293T cells from transfected plasmids and then passaged each virus in triplicate in chicken eggs, using a dose of 10^4^ EID_50_ (based on allantois-on-shell infectivity) to initiate each passage. We harvested each passage at 16 hours post-infection (h.p.i.) to minimize defective interfering particle formation. After the third passage, we sequenced the entire HA coding region of all six passaged populations, along with the parental seed stocks, using an optimized primerID protocol, (see Methods). To overcome the sequence read length limitations of the Illumina Miseq platform (<550nt read length limit), we used four amplicons to cover the entire HA coding region.

**Fig 5:**
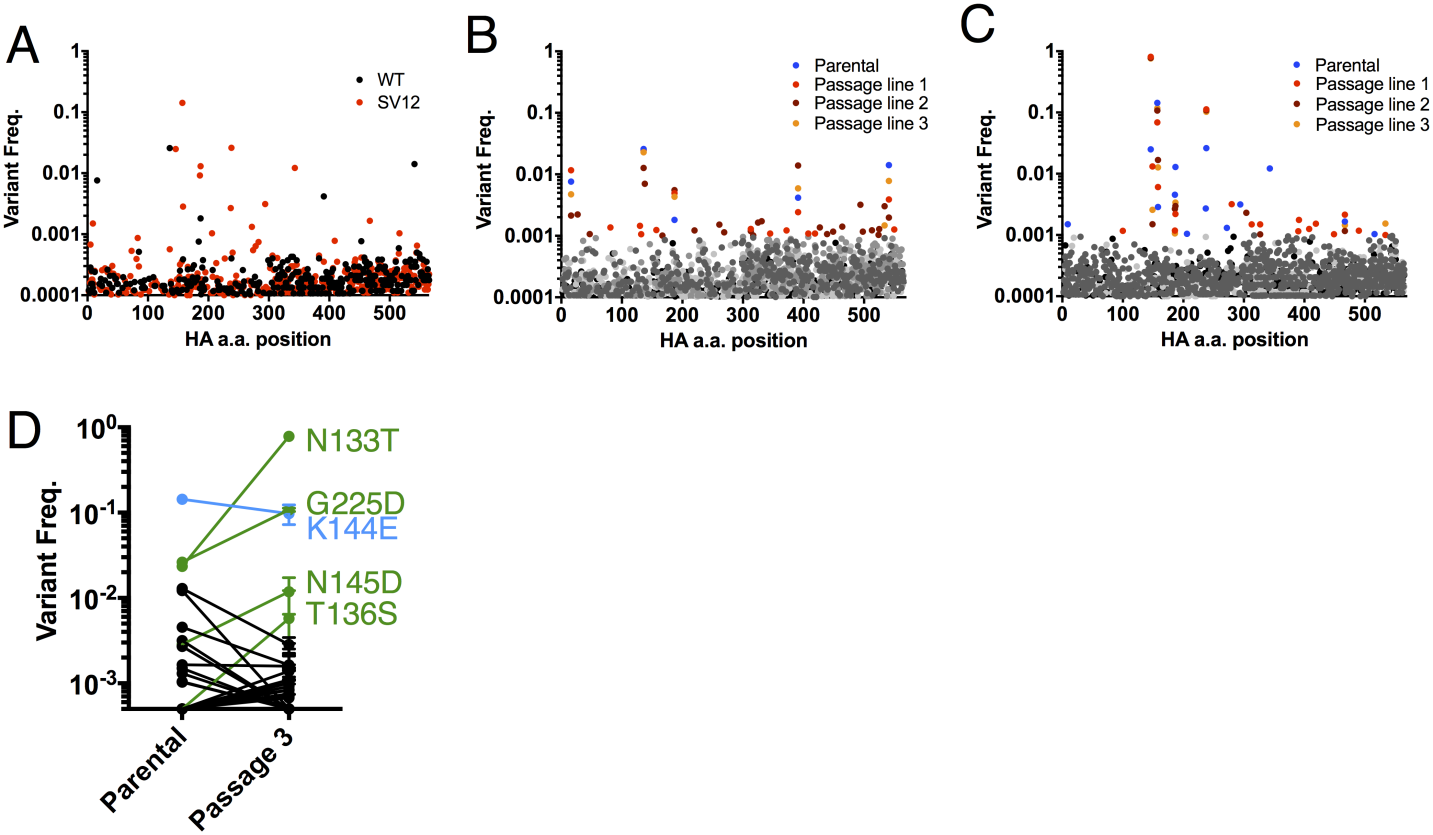
PrimerID sequencing of HA genes from recombinant WT and SV12-HA following passage. Recombinant PR8 (WT) and rSV12-HA (SV12) viruses were rescued via reverse genetics. **(A)** Amino acid variant frequencies within WT (black) and SV12 HA (red) populations collected from rescue supernatants without amplification, as determined by primerID sequencing. (**B,C**) Amino acid variant frequencies within WT **(B)** and rSV12-HA **(C)** populations following three passages in eggs. For each population, all variants over 0.1% are colored to allow them to be visually distinguished. **(D)** Comparison of amino acid variant frequencies within rSV12-HA populations before and after 3 passages in eggs. Passage 3 data represents mean +/- SD of three independent passage lines. All amino acid numbering indicated on graphs **A-C** is from initiating methionine, not according to H3 system.

Both SV12 and PR8 (WT) parental populations harbored numerous missense mutations at frequencies above background in HA (Fig 5A). Five such mutations resulted in amino acid substitutions that were present in the WT PR8 parental population at frequencies over 0.1%, with a K123T substitution dominant at 2.5% (Fig 5B). Four of the WT variants likely emerged due to stochastic genetic drift resulting from bottlenecking during the rescue process, as indicated by minimal change in frequency by passage 3 [27]. One variant enriched in the WT parental population, K174E, increased slightly in frequency in all three passage lines, and may represent a tissue culture adaptive variant as it was also present in the parental rSV12-HA population. No other variants exhibited a consistent pattern of emergence during passaging of the WT populations.

By contrast, the parental rSV12-HA population carried nearly three times as many missense mutations (14 vs. 5) at frequencies >0.1% (Fig 5C), primarily located in the globular domain of HA. Only K174E was also present in the WT parental population. Five of the 14 variants increased or were maintained at high frequency during passaging in all three SV12 populations: N133T, T136S, K144E, N145D, and G225D (Fig 5D). Of these, N133T exhibited the most dramatic increase in frequency and approached fixation in all three passage lines. Strikingly, four of the five variants carried substitutions at residues that had elevated variant frequencies in our first experiment: N133, K144, N145, and G225, indicating that compensation for antigenic escape can follow predictable genetic pathways under highly controlled conditions.

### Linkage analysis reveals near mutual exclusivity among compensatory substitutions during passage

Emergence of an amino acid variant within a population may result from positive selection driven by specific beneficial effects of the variant on fitness. Alternatively, the variant may be neutral or deleterious but hitchhike with another variant under positive selection to attain higher frequency. The potential for intragenic hitchhiking is high with IAV due to extreme rarity of intragenic recombination during infection [28]. PrimerID sequencing allows the direct quantification of linkage between amino acid positions present in individual viral cDNAs, thereby defining the relative contributions of direct selection *vs*. hitchhiking in amino acid variant emergence.

**Fig 6:**
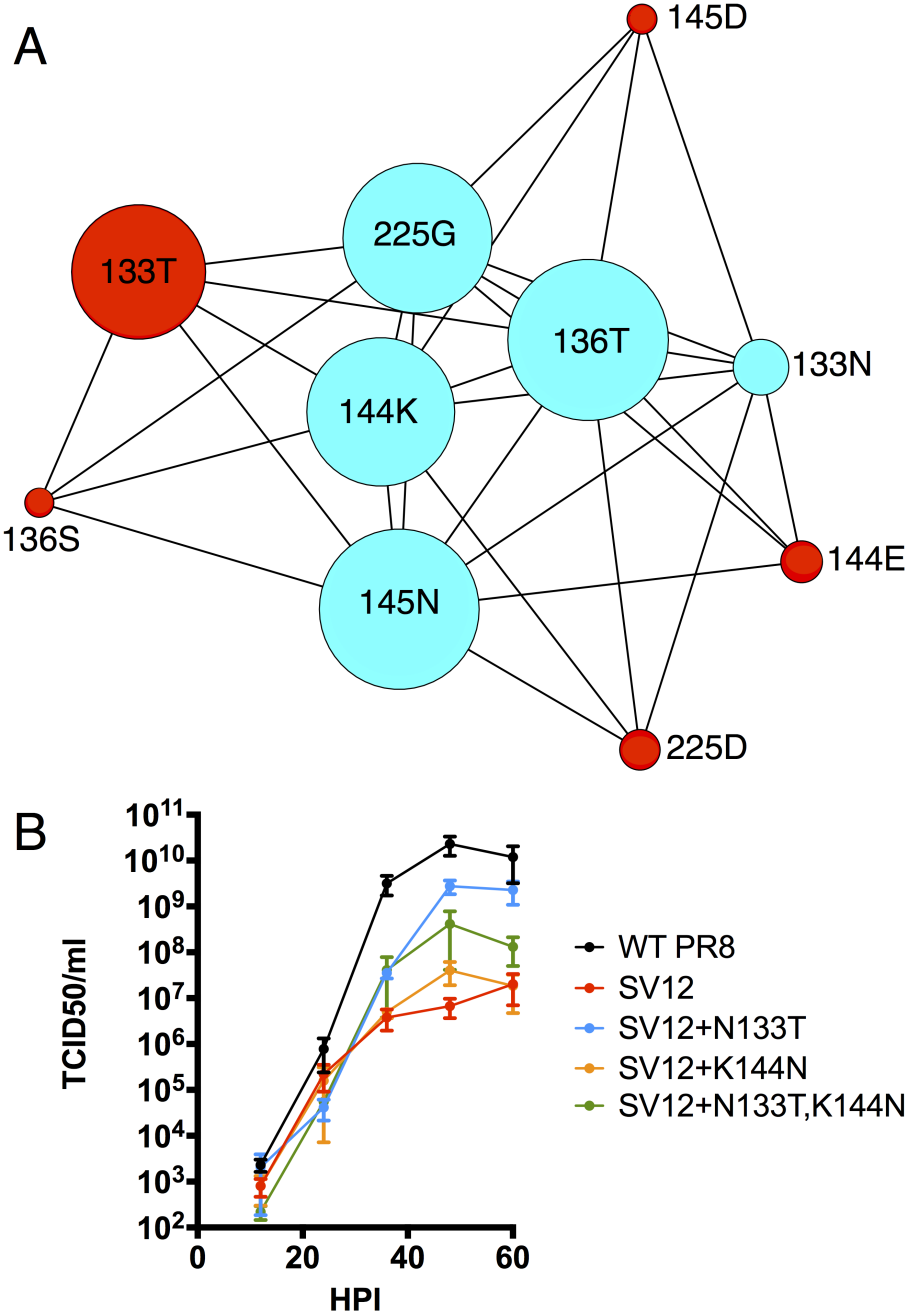
Linkage analysis of high frequency variants that emerged during rSV12-HA passage. **(A)** Graphical representation of genetic linkage between selected variants within the three parallel passage 3 rSV12-HA populations, based on PrimerID sequencing. Node size is proportional to relative frequency of the indicated allele across the three populations. Aqua nodes indicate parental amino acids and red nodes represent variant amino acids. Edges connect pairs of amino acids that were linked in greater than 0.02% of all primerID consensus reads examined. **(B)** Multi-step growth comparison of the indicated viruses in eggs.

We assessed the extent of genetic linkage between the five high frequency variants that emerged in all three rSV12-HA passage populations (N133T, T136S, K144E, N145D, and G225D) (Fig 5D). Examination of linkage at passage 3 revealed that 4 of the variants, N133T, K144E, N145D, and G225D, were mutually exclusive within single HA variants (with just a handful of exceptions) (Fig 6A, S3 and S4 Tables). In contrast, T136S was only observed as paired with N133T. These results were consistent with the nearly mutually exclusive relationships determined for the substitutions observed in the first sequencing experiment carried out on the original SV12 stock (Fig 2, S2 Table).

These findings suggest that each emergent, mutually-exclusive substitution was selected individually for its ability to compensate for the fitness defects of SV12, and that their combination in the same variant may substantially reduce fitness. The data, however, are also consistent with the predicted infrequent recombination within IAV genome segments and a strong role for clonal interference in governing the emergence of beneficial alleles during adaptation [29,30].

To test whether combining compensatory HA glycan additions decreases viral fitness, we generated a N133T, K144N double mutant (rSV12(N133T, K144N)) using reverse genetics. After confirming the double mutant did incorporate an additional glycan compared to the single mutants by SDS-PAGE (S3 Fig), we examined its growth in eggs. The double mutant achieved significantly higher titers than rSV12-HA, but lower than rSV12(N133T) (Fig 6B). This result can be explained by the binding activity of the double mutant, which is comparable to rSV12(N133T) based on BLI (Table 1). Together, these data suggest that clonal interference rather than reduced fitness may be the primary explanation for the mutual exclusivity of compensatory substitutions that we observe.

## Discussion

IAV has persisted in humans despite widespread vaccination and infection, while vaccination has driven other RNA viruses with similarly high mutation rates, such as measles virus and poliovirus, to near-extinction. Understanding the specific features of IAV that enable effective immune escape is critical for designing more effective vaccines and therapeutics.

Our findings reveal a novel role for glycosylation in viral immune evasion, independent of its well-known steric effects on epitope shielding [14–19]. Previous studies clearly showed that glycan addition generally impairs HA function while dramatically increasing fitness in the presence of neutralizing antibodies [5]. Our results demonstrate that glycan addition can also play the opposite role during antigenic drift: increasing the fitness of antigenic escape variants by restoring optimal HA receptor interactions, even in the absence of antibody selective pressure. This raises the intriguing possibility that both mechanisms contribute to the steady evolutionary accretion of N-linked glycans within the globular domain observed during human circulation of H1 and H3 viruses [17].

Importantly, the fact that we selected for glycan addition in the absence of Ab selection does not diminish the profound effects that these glycans have on the antigenicity of HA. In particular, the predominant variant selected, N133S/T, also emerged during the circulation of seasonal H1N1 in humans, and has been demonstrated to have enormous effects on antigenicity and sensitivity to neutralization [17]. These results highlight the self-perpetuating nature of HA antigenic evolution. Hensley et al. found that infection of naïve hosts with antigenic escape variants selected for compensatory mutations that were themselves antigenically significant [13]. Similarly, our results demonstrate that antigenic escape can lead to the emergence of compensatory mutations that push HA even further in antigenic space. These factors make it difficult to infer the nature of selective pressures resulting in given HA sequence changes in human IAV isolates.

Defining the specific properties of the HA molecule that facilitate mutational tolerance is an area of intense study. Studies *in vitro* found that the HA globular head was highly tolerant of both random substitutions and 5-amino acid block insertions [3,27,31,32]. These results suggested that the HA globular head may exhibit a high degree of mutational robustness, such that the median fitness effects of substitutions are relatively small [32–34]. Alternatively, mutations may generally be costly, but are compensated by epistatic mutations that restore protein function and fitness[6,35]. Our data, along with other studies that demonstrate fitness costs associated with antigenic variation and/or rapid emergence of compensatory mutations following HA antigenic escape, suggest that compensatory epistatic interactions play a critical role during antigenic drift [6,13,36,37].

If rapid compensation of antigenic escape mutations is important for maintaining fitness during antigenic drift, then defining compensatory mechanisms is essential. Many antigenicity-determining residues scattered in the HA globular domain also strongly influence avidity and specificity for sialic acid receptors, and thus Ab escape substitutions at these residues can negatively affect receptor interactions[13]. As a result, many of the compensatory mutations that have been described in association with antigenic escape affect receptor interactions, and likely serve to restore optimal receptor binding properties. The need to accumulate compensatory mutations in order to combine antigenic novelty with fitness and transmissibility has been proposed as an explanation for the discordance between the high *in vitro* mutation rates and rapid Ab-based selection of influenza viruses *in vivo* and the relatively slow rates of population-level antigenic evolution seen with influenza viruses[13,38]. Our results indicate that glycosylation can play an important role in this process.

The availability of multiple mutational pathways to restore fitness increases the odds that a fit variant will emerge under selection [39]. Our discovery that glycan addition beneficially tunes receptor interactions adds to previously described mechanisms, including charge transitions in the globular HA domain and alterations in NA activity [13,21,36,40]. While difficult to test experimentally, it is intriguing to consider that IAV may have evolved to maximize the number of compensatory pathways available during antigenic drift.

Our unexpected discovery of a new role for glycosylation in compensating for IAV immune evasion highlights the power of virus population sequencing for uncovering novel mechanisms of viral adaptation through high-definition forward genetics screening. The ability to accurately measure changes in the viral population through methods such as primerID can provide a comprehensive, unbiased profile of the genetic pathways to increased fitness under defined selection conditions.

The primerID approach offers several advantages over conventional deep sequencing protocols. *First*, the ability to generate a multi-read consensus of the original cDNA sequence reduces the background nucleotide error rate from ~1% to ~0.01%, greatly increasing the sensitivity of minor variant detection. In theory, this background represents the *bona fide* mutation frequency within the population, combined with the mutation rate of the reverse transcriptase used to generate the cDNA pool (~0.01%)[23]. *Second*, assembly of amplicon reads into ancestral cDNA sequences eliminates the distortion of measured variant frequencies by the stochastic PCR re-sampling that occurs during conventional amplicon sequencing. *Third*, the number of unique primerID barcodes used to generate consensus sequences represents the actual depth of the viral population sequenced. *Fourth*, the ability to phase substitutions into authentic haplotypes enables quantitation of linkage between substitutions within a given genome segment. Finally, primerID requires far less virus input than other methods of highly accurate population sequencing such as CirSeq, facilitating a wider range of experimental applications, including the analysis of samples collected from infected animals or human subjects [41,42].

Altogether, we reveal a surprising new role for glycosylation in facilitating IAV immune evasion by compensating for the fitness costs of antigenic escape mutations. These results broaden our understanding of the critical function of glycosylation during antigenic drift and shed light on the unique mutational plasticity of influenza HA. Further, these studies highlight the remarkable potential of highly accurate virus population sequencing and high-definition forward genetics for exploring viral evolution.

## Materials and methods

### Ethics

We obtained chicken eggs from a commercial vendor.

### Viruses

The original SV12 stock was generated as previously described [21], and passaged 2–3 times in eggs in the absence of antibodies prior to RNA extraction and sequencing. We generated the A/Puerto Rico/8/1934 (PR8) strain using the eight-plasmid rescue system (plasmids generously provided by Dr. Adolfo Garcia-Sastre; Icahn School of Medicine at Mt. Sinai, New York). We generated seed virus by cotransfecting 293T cells (obtained from ATCC) with the eight gene segment-encoding plasmids. Seed virus stocks were expanded once in 10-day-old embryonated, specific pathogen-free (SPF) eggs. Allantoic fluid was collected 48 hours post infection, and clarified by centrifugation. A reverse genetic construct expressing the consensus HA gene segment sequence from SV12 was generated by RT-PCR amplification of the HA-encoding segment from SV12 virions, and cloning it into the pDZ vector. Virus mutants were generated by site-directed PCR mutagenesis of the relevant reverse genetics constructs. All infectious virus titers were determined by end-point dilution on MDCK cells (obtained from ATCC) in Gibco minimal essential medium (MEM) supplemented with 1 μg/mL trypsin treated with l-(tosylamido-2-phenyl) ethyl chloromethyl ketone (TPCK-treated trypsin), 1mM HEPES buffer (Corning), and gentamycin. TCID_50_ titers were determined using the Reed–Muench method.

### Comparison of viral growth kinetics

To compare peak titers of molecular clone-derived mutants, 500 TCID_50_ of each virus were inoculated into 10 day old embryonated eggs in triplicate. Allantoic fluid was collected 48 hours post infection, and titers determined based on end-point dilution using MDCK cells as described above.

### HA glycan analysis

MCDK cells were infected with rSV12-HA and each molecular clone-derived mutant at an MOI of 5 TCID_50_/cell. After 1 hour, virus supernatant was removed and replaced with Gibco MEM supplemented with 7.5% fetal bovine serum, after washing cells with PBS containing CaCl_2_ and MgCl_2_. Six hours post infection, cells were washed twice with PBS, and then exposed to lysis buffer containing 1% sodium dodecyl sulfate, 50mM Tris-HCl pH 7.5, 10mM dithiothreitol (DTT), 15U/mL DNase1, and mini-complete protease inhibitor. Lysate was then digested either with EndoH or PNGase F or left untreated. Control or digested lysates were then immunoblotted using the mouse HA2 chain specific mAb RA5-22[43].

### Passaging experiments

Recombinant PR8 and SEQ12HA viruses were serially passaged three times in 10-day old embryonated chicken eggs (Charles River) at a titer of 10^4^ AOS ID/egg at 35°C. At 16 hours post infection, infected allantoic fluid was harvested, clarified and titrated using the AOS method for the subsequent passage [44].

### PrimerID library preparation

In experiments using recombinant viruses, hemagglutinin was sequenced using four overlapping amplicons spanning the entire coding sequence. Viral RNA was extracted from allantoic fluid using the QiaAmp Viral RNA Mini Kit. Viral RNA was reverse transcribed with Accuscript Hi-Fi Reverse Transcriptase (Agilent) with the following primers bearing random 12-mer barcodes (All Ns hand-mixed to achieve ~25:25:25:25 ratio): PR8_SV12 Amp 1F 5’-ACACTCTTTCCCTACACGACGCTCTTCCGATCTNNNNNNNNNNNNCAGGAAAATAAAAACAACCAAA ATG-3’, PR8_SV12 Amp 2F 5’-ACACTCTTTCCCTACACGACGCTCTTCCGATCTNNNNNNNNNNNNCACCAAAGAAAGCTCATGGCCC-3’, PR8_SV12 Amp 3F 5’-ACACTCTTTCCCTACACGACGCTCTTCCGATCTNNNNNNNNNNNNCAACACGAAGTGTCAAACACCC -3’, PR8_SV12 Amp 4F 5’-ACACTCTTTCCCTACACGACGCTCTTCCGATCTNNNNNNNNNNNNCAGGATTTCTGGACATTTGGAC -3’. The reverse transcription reactions were purified using the PureLink PCR Purification kit (Invitrogen) using the high-molecular-weight cutoff buffer. First-round PCR was performed using Platinum Taq DNA Polymerase High Fidelity (Invitrogen) with an estimated 10^5^ copies of cDNA templates, a universal forward primer (5’-AATGATACGGCGACCACCGAGATCTACACTCTTTCCCTACAC-3’) and amplicon-specific reverse primers: PR8_SV12 Amp 1R 5’-GTGACTGGAGTTCAGACGTGTGCTCTTCCGATCTNNNNATGGGAGCATGCTGCCGTTA-3’, PR8_SV12 Amp 2R 5’-GTGACTGGAGTTCAGACGTGTGCTCTTCCGATCTNNNNGGGAGACTGCTGTTTATAGC-3’, PR8_SV12 Amp 3R 5’-GTGACTGGAGTTCAGACGTGTGCTCTTCCGATCTNNNNCAGAGTCCTTTCATTTTCCA-3’, PR8_SV12 Amp 4R 5’-GTGACTGGAGTTCAGACGTGTGCTCTTCCGATCTNNNNATATCTCTGAAATTCTAATC-3’.

Sample indices were finally added using KAPA HiFi HotStart PCR Kit, 2.5μL of the first round PCR products and indexed primers (5’- CAAGCAGAAGACGGCATACGAGAT(8-mer sample ID)GTGACTGGAGTTCAGACGTGTG-3’). Amplicons were gel purified using the Qiagen Gel Extraction Kit and quantified on a Nanodrop 1000. Amplicons were pooled into a single library, quantified with the KAPA Library Quantification Kit, and sequenced using the MiSeq Reagent Kit V3 with 325x325 paired-end cycles. The raw sequencing data is available at the NCBI Short Read Archive under accession numbers SRR5428773-SRR5428801.

The primerID library preparation methods used for the original SV12 stock (Fig 2) differed from the optimized pipeline described above in several substantive ways. A single amplicon spanning HA amino acids 115 to 258 was generated using an RT primer with a random 10-mer barcode and the Superscript III RT enzyme (Thermo Fisher). Paired-end reads of the amplicon did not overlap, leaving a gap involving codons 184–186. RT primer (HAF381): ACACTCTTTCCCTACACGACGCTCTTCCGA TCTNNNNNNNNNNCAGCTAAGAGAGCAATTGAGCTCAGTGTCATC. Reverse PCR primer HAr813: GTGACTGGAGTTCAGACGTGTGCTCTTCCGATCTNNNN GATGCCGGACCCAAAGCCTCTACTCAGTGC. Subsequent library preparation was as outlined above.

### PrimerID data analysis

For the sequence analysis of passaging experiments using the recombinant PR8 and SV12 viruses, an optimized analysis pipeline was employed. In this pipeline, the first 4 bases of R2 reads were trimmed using fastx_trimmer from the FASTX Toolkit (v. 0.0.13; http://hannonlab.cshl.edu/fastx_toolkit/). Overlapping paired-end reads were merged into a single read using PANDAseq (v. 2.9)[45]. Merged reads were processed using filter_fastq_by_primerid_length.pl to identify and extract the primerID from each read, with options—post CA—removepost, and place the primerID sequence in the sequence id line for each read. Each FASTQ file was split into separate amplicon libraries using Btrim64[46] with options -u 2 -v 2 -S -B -e 300; a list of primers for each amplicon was also supplied. Off-target sequences were removed with get_majority_block_bam.pl, a wrapper script that maps the reads using BWA MEM (v. 0.7.12-r1039[47] with options -B 1 -M, converts the output to BAM using Picard SortSam (v. 1.130; http://broadinstitute.github.io/picard/), and retains only the reads that have the most common mapped start and stop coordinates using BEDTools (v. 2.19.1)[48]. Reads were collapsed into consensus sequences using merge_primerid_read_groups.pl with options—ambig 600—min_freq 0.75 to require that the consensus base called makes up at least 75% of the bases for that position within the read group, otherwise an ambiguous base is called. The minimum group size (-m) to use in the merging process was computed for each amplicon within each sample (i.e., each library), using a formula derived for 12 bp primerID length, based on the simulation model proposed by Zhou et al.[23] in order to reduce the effect of offspring primerID reads from large primerID groups on smaller primerID groups. (To derive the formula for 12 bp primerIDs, the script consensus_cutoff.rb was run with length_of_primer_id set to 12.) If the computed minimum group size was less than 5, then -m was set to 5. Consensus reads were converted to frequency tables for nucleotides, codons, and amino acids at each position within the amplicon using convert_reads_to_amino_acid.pl.

Sequence analysis of the original SV12 stock library (Fig 2), generated using a slightly different barcode primer design, substantively differed from the optimized analysis approach described above as follows: PrimerID groups required a minimum of 2 reads, and the intra-sample consensus was used as a tiebreaker.

To determine linkage between high frequency variants (MAF > 0.5%), we performed a Fisher’s exact test for every pair of variants within each replicate and we derived a combined p-value from these individual replicate p-values using Fisher’s method.

All software used for the optimized primerID analysis is available on Github: http://github.com/niaid/primer-id-progs.

#### PrimerID-ViVan comparison

Libraries for the parental and passaged viruses were constructed as previously described. Briefly, full-length genomes were amplified from purified viral RNA using a multisegment RT-PCR protocol[49]. Illumina libraries were then constructed using the Nextera XT DNA Library Prep Kit (Illumina) and sequenced on the Illumina MiSeq platform according the manufacturer’s protocols. Variants for each library were then called with the ViVan pipeline[24]. Variant frequencies were then compared between primer ID and ViVan calls which passed filter.

#### Receptor binding analysis

We used Streptavidin Dip and Read Biosensors (ForteBIO, Menlo Park, CA, USA) to measure binding of viruses to sialic acid. Graded amounts of biotinylated 3’-Sialyl lactosamine PAA-biotin aka 3’SLN (GlykoTech) were captured on an avidin coated biosensor. Virus binding experiments were performed by an Octet QKe biolayer interferometer (ForteBio). All of the viruses were diluted in PBS, pH = 7.4, 0.01% BSA, 0.002% Tween-20, (kinetic buffer) for binding to 6’SLN to 1pM in presence of 10μM Oseltamivir (ARC, St. Louis, Mo) and Zanamivir (Moravek Biochemicals, Brea,CA) to block viral NA activity. Binding of virus was measured at 30°C for 10 min followed by a 10 min dissociation measurement.

## Supporting information

S1 Fig Variant calling comparison between ViVan and primerID methods. HA libraries were prepared for both Nextera and amplicon sequencing, and analyzed with ViVan and PrimerID pipelines, respectively. Variant frequencies were compared between the final outputs of both methods for parental wild-type and sequential 12 viruses **(A and B)** and all common variants in three replicates for passage 3 wild-type and sequential 12 viruses **(C and D)**. Red line indicates the expected 1:1 ratio.
(TIFF)

S2 Fig Comparison of background variant frequency levels between ViVan and primerID methods. (**A, B**) Combined minor amino acid variant frequency is shown for each position of HA amplicon 2 region in SV12 parental sample for unmerged reads (before applying PrimerID), PrimerID consensus reads, and Nextera shotgun sequencing, evaluated by ViVan. **(B)** is a zoomed-in view of **(A)**. (**C, D**) Fold enrichment of variant frequency over median variant frequency for the amplicon region, shown for the same three methods in **(A)** and **(B)**. **(D)** is a zoomed-in view of **(C)**.
(TIF)

S3 Fig Non-reducing WB analysis of double glycan addition mutant. Immunoblot of purified virions as indicated electrophoresed under non-reducing conditions using the HA2 specific mAb RA5-22.
(TIFF)

S1 Table PrimerID noise reduction. (TIF)

S2 Table Minor allele frequency background level in primerID and Nextera/ViVan Sequencing. (TIFF)

S3 Table Pairwise amino acid variant combinations within primerID consensus reads of the original SV12 stock. Variant amino acids were evaluated pairwise to determine the frequency at which any two substitutions were observed in the same primerID consensus read. The combined counts across the two biological replicates that were sequenced are listed for each pairwise amino acid variant combination.
(TIFF)

S4 Table Specific amino acid substitution combinations observed within primer ID consesus reads in parental and passage 3 recombinant rSV12-HA populations. Each primerID consensus read was assessed for its inferred amino acid identity at positions 133, 136, 144, 145, and 225 (wild type at each position is NTKNG, respectively). The number of primerID consesus reads that contained the indicated amino acid identities at these positions is listed. Variant amino acids are highlighted in red, and combinations that contain two variant amino acids are in bold face. This table only displays amino acid variant combinations that were observed in at least two primerID concensus reads across all populations examined.
(TIFF)

S5 Table Statistical assessment of linkage between high frequency amino acid substitutions that emerged during rSV12-HA passage. Combined p-values from three replicate populations as determined by Fisher’s method.
(TIFF)

## References

[1] Wiley DC, Wilson IA, Skehel JJ. Structural identification of the antibody-binding sites of Hong Kong influenza haemagglutinin and their involvement in antigenic variation. Nature. 1981 1 29;289(5796):373–8. 6162101 10.1038/289373a0

[2] Caton AJ, Brownlee GG, Yewdell JW, Gerhard W. The antigenic structure of the influenza virus A/PR/8/34 hemagglutinin (H1 subtype). Cell. 1982 12;31(2 Pt 1):417–27. 6186384 10.1016/0092-8674(82)90135-0

[3] Thyagarajan B, Bloom JD. The inherent mutational tolerance and antigenic evolvability of influenza hemagglutinin. eLife. 2014;3. 10.7554/eLife.03300 PMC4109307 25006036

[4] Angeletti D, Gibbs JS, Angel M, Kosik I, Hickman HD, Frank GM, et al Defining B cell immunodominance to viruses. Nat Immunol. 2017 2 13;18(4):456–63. doi: 10.1038/ni.3680 28192417 10.1038/ni.3680 PMC5360521

[5] Das SR, Hensley SE, David A, Schmidt L, Gibbs JS, Puigbò P, et al Fitness costs limit influenza A virus hemagglutinin glycosylation as an immune evasion strategy. Proc Natl Acad Sci U S A. 2011 12 20;108(51):E1417–1422. doi: 10.1073/pnas.1108754108 22106257 10.1073/pnas.1108754108 PMC3251056

[6] Kryazhimskiy S, Dushoff J, Bazykin GA, Plotkin JB. Prevalence of epistasis in the evolution of influenza A surface proteins. PLoS Genet. 2011 2;7(2):e1001301 doi: 10.1371/journal.pgen.1001301 21390205 10.1371/journal.pgen.1001301 PMC3040651

[7] Yewdell JW, Taylor A, Yellen A, Caton A, Gerhard W, Bächi T. Mutations in or near the fusion peptide of the influenza virus hemagglutinin affect an antigenic site in the globular region. J Virol. 1993 2;67(2):933–42. 7678310 10.1128/jvi.67.2.933-942.1993 PMC237447

[8] Hawman DW, Fox JM, Ashbrook AW, May NA, Schroeder KMS, Torres RM, et al Pathogenic Chikungunya Virus Evades B Cell Responses to Establish Persistence. Cell Rep. 2016 8 2;16(5):1326–38. doi: 10.1016/j.celrep.2016.06.076 27452455 10.1016/j.celrep.2016.06.076 PMC5003573

[9] Goo L, VanBlargan LA, Dowd KA, Diamond MS, Pierson TC. A single mutation in the envelope protein modulates flavivirus antigenicity, stability, and pathogenesis. PLoS Pathog. 2017 2;13(2):e1006178 doi: 10.1371/journal.ppat.1006178 28207910 10.1371/journal.ppat.1006178 PMC5312798

[10] Fazekas S, Groth S. Antigenic, Adaptive and Adsorptive Variants of the Influenza a Hemagglutinin. In: Laver WG, Bachmayer H, Weil R, editors. The Influenza Virus Hemagglutinin: Symposium, Baden near Vienna, March 21–23, 1977 [Internet]. Vienna: Springer Vienna; 1978. p. 25–48. http://dx.doi.org/10.1007/978-3-7091-4130-4_3

[11] Yewdell JW, Caton AJ, Gerhard W. Selection of influenza A virus adsorptive mutants by growth in the presence of a mixture of monoclonal antihemagglutinin antibodies. J Virol. 1986 2;57(2):623–8. 2418215 10.1128/jvi.57.2.623-628.1986 PMC252777

[12] Temoltzin-Palacios F, Thomas DB. Modulation of immunodominant sites in influenza hemagglutinin compromise antigenic variation and select receptor-binding variant viruses. J Exp Med. 1994 5 1;179(5):1719–24. 8163950 10.1084/jem.179.5.1719 PMC2191490

[13] Hensley SE, Das SR, Bailey AL, Schmidt LM, Hickman HD, Jayaraman A, et al Hemagglutinin receptor binding avidity drives influenza A virus antigenic drift. Science. 2009 10 30;326(5953):734–6. doi: 10.1126/science.1178258 19900932 10.1126/science.1178258 PMC2784927

[14] Skehel JJ, Stevens DJ, Daniels RS, Douglas AR, Knossow M, Wilson IA, et al A carbohydrate side chain on hemagglutinins of Hong Kong influenza viruses inhibits recognition by a monoclonal antibody. Proc Natl Acad Sci U S A. 1984 3;81(6):1779–83. 6584912 10.1073/pnas.81.6.1779 PMC345004

[15] Wei X, Decker JM, Wang S, Hui H, Kappes JC, Wu X, et al Antibody neutralization and escape by HIV-1. Nature. 2003 3 20;422(6929):307–12. doi: 10.1038/nature01470 12646921 10.1038/nature01470

[16] O’Donnell CD, Wright A, Vogel LN, Wei C-J, Nabel GJ, Subbarao K. Effect of priming with H1N1 influenza viruses of variable antigenic distances on challenge with 2009 pandemic H1N1 virus. J Virol. 2012 8;86(16):8625–33. doi: 10.1128/JVI.00147-12 22674976 10.1128/JVI.00147-12 PMC3421718

[17] Medina RA, Stertz S, Manicassamy B, Zimmermann P, Sun X, Albrecht RA, et al Glycosylations in the globular head of the hemagglutinin protein modulate the virulence and antigenic properties of the H1N1 influenza viruses. Sci Transl Med. 2013 5 29;5(187):187ra70 doi: 10.1126/scitranslmed.3005996 23720581 10.1126/scitranslmed.3005996 PMC3940933

[18] Helle F, Goffard A, Morel V, Duverlie G, McKeating J, Keck Z-Y, et al The neutralizing activity of anti-hepatitis C virus antibodies is modulated by specific glycans on the E2 envelope protein. J Virol. 2007 8;81(15):8101–11. doi: 10.1128/JVI.00127-07 17522218 10.1128/JVI.00127-07 PMC1951279

[19] Lennemann NJ, Rhein BA, Ndungo E, Chandran K, Qiu X, Maury W. Comprehensive functional analysis of N-linked glycans on Ebola virus GP1. mBio. 2014;5(1):e00862–00813. doi: 10.1128/mBio.00862-13 24473128 10.1128/mBio.00862-13 PMC3950510

[20] Underwood PA, Skehel JJ, Wiley DC. Receptor-binding characteristics of monoclonal antibody-selected antigenic variants of influenza virus. J Virol. 1987 1;61(1):206–8. 3783824 10.1128/jvi.61.1.206-208.1987 PMC255240

[21] Das SR, Hensley SE, Ince WL, Brooke CB, Subba A, Delboy MG, et al Defining influenza A virus hemagglutinin antigenic drift by sequential monoclonal antibody selection. Cell Host Microbe. 2013 3 13;13(3):314–23. doi: 10.1016/j.chom.2013.02.008 23498956 10.1016/j.chom.2013.02.008 PMC3747226

[22] Jabara CB, Jones CD, Roach J, Anderson JA, Swanstrom R. Accurate sampling and deep sequencing of the HIV-1 protease gene using a Primer ID. Proc Natl Acad Sci U S A. 2011 12 13;108(50):20166–71. doi: 10.1073/pnas.1110064108 22135472 10.1073/pnas.1110064108 PMC3250168

[23] Zhou S, Jones C, Mieczkowski P, Swanstrom R. Primer ID Validates Template Sampling Depth and Greatly Reduces the Error Rate of Next-Generation Sequencing of HIV-1 Genomic RNA Populations. J Virol. 2015 8;89(16):8540–55. doi: 10.1128/JVI.00522-15 26041299 10.1128/JVI.00522-15 PMC4524263

[24] Isakov O, Borderia AV, Golan D, Hamenahem A, Celniker G, Yoffe L, et al Deep sequencing analysis of viral infection and evolution allows rapid and detailed characterization of viral mutant spectrum. Bioinformatics. 2015 7 1;31(13):2141–50. doi: 10.1093/bioinformatics/btv101 25701575 10.1093/bioinformatics/btv101 PMC4481840

[25] McCrone JT, Lauring AS. Measurements of Intrahost Viral Diversity Are Extremely Sensitive to Systematic Errors in Variant Calling. Dermody TS, editor. J Virol. 2016 8 1;90(15):6884–95. doi: 10.1128/JVI.00667-16 27194763 10.1128/JVI.00667-16 PMC4944299

[26] Aebi M, Bernasconi R, Clerc S, Molinari M. N-glycan structures: recognition and processing in the ER. Trends Biochem Sci. 2010 2;35(2):74–82. doi: 10.1016/j.tibs.2009.10.001 19853458 10.1016/j.tibs.2009.10.001

[27] Doud M, Bloom J. Accurate Measurement of the Effects of All Amino-Acid Mutations on Influenza Hemagglutinin. Viruses. 2016 6 3;8(6):155. 10.3390/v8060155 PMC4926175 27271655

[28] Boni MF, Zhou Y, Taubenberger JK, Holmes EC. Homologous Recombination Is Very Rare or Absent in Human Influenza A Virus. J Virol. 2008 5 15;82(10):4807–11. doi: 10.1128/JVI.02683-07 18353939 10.1128/JVI.02683-07 PMC2346757

[29] Miralles R. Clonal Interference and the Evolution of RNA Viruses. Science. 1999 9 10;285(5434):1745–7. 10481012 10.1126/science.285.5434.1745

[30] Strelkowa N, Lässig M. Clonal interference in the evolution of influenza. Genetics. 2012 10;192(2):671–82. doi: 10.1534/genetics.112.143396 22851649 10.1534/genetics.112.143396 PMC3454888

[31] Heaton NS, Sachs D, Chen C-J, Hai R, Palese P. Genome-wide mutagenesis of influenza virus reveals unique plasticity of the hemagglutinin and NS1 proteins. Proc Natl Acad Sci U S A. 2013 12 10;110(50):20248–53. doi: 10.1073/pnas.1320524110 24277853 10.1073/pnas.1320524110 PMC3864309

[32] Visher E, Whitefield SE, McCrone JT, Fitzsimmons W, Lauring AS. The Mutational Robustness of Influenza A Virus. PLoS Pathog. 2016 8;12(8):e1005856 doi: 10.1371/journal.ppat.1005856 27571422 10.1371/journal.ppat.1005856 PMC5003363

[33] de Visser JAGM, Hermisson J, Wagner GP, Ancel Meyers L, Bagheri-Chaichian H, Blanchard JL, et al Perspective: Evolution and detection of genetic robustness. Evol Int J Org Evol. 2003 9;57(9):1959–72. 10.1111/j.0014-3820.2003.tb00377.x 14575319

[34] Lauring AS, Frydman J, Andino R. The role of mutational robustness in RNA virus evolution. Nat Rev Microbiol. 2013 5;11(5):327–36. doi: 10.1038/nrmicro3003 23524517 10.1038/nrmicro3003 PMC3981611

[35] Gong LI, Suchard MA, Bloom JD. Stability-mediated epistasis constrains the evolution of an influenza protein. eLife. 2013;2:e00631 doi: 10.7554/eLife.00631 23682315 10.7554/eLife.00631 PMC3654441

[36] Hensley SE, Das SR, Gibbs JS, Bailey AL, Schmidt LM, Bennink JR, et al Influenza A virus hemagglutinin antibody escape promotes neuraminidase antigenic variation and drug resistance. PloS One. 2011;6(2):e15190 doi: 10.1371/journal.pone.0015190 21364978 10.1371/journal.pone.0015190 PMC3043005

[37] Neverov AD, Kryazhimskiy S, Plotkin JB, Bazykin GA. Coordinated Evolution of Influenza A Surface Proteins. PLoS Genet. 2015 8;11(8):e1005404 doi: 10.1371/journal.pgen.1005404 26247472 10.1371/journal.pgen.1005404 PMC4527594

[38] Koelle K, Cobey S, Grenfell B, Pascual M. Epochal evolution shapes the phylodynamics of interpandemic influenza A (H3N2) in humans. Science. 2006 12 22;314(5807):1898–903. doi: 10.1126/science.1132745 17185596 10.1126/science.1132745

[39] Weinreich DM, Delaney NF, Depristo MA, Hartl DL. Darwinian evolution can follow only very few mutational paths to fitter proteins. Science. 2006 4 7;312(5770):111–4. doi: 10.1126/science.1123539 16601193 10.1126/science.1123539

[40] Brooke CB, Ince WL, Wei J, Bennink JR, Yewdell JW. Influenza A virus nucleoprotein selectively decreases neuraminidase gene-segment packaging while enhancing viral fitness and transmissibility. Proc Natl Acad Sci U S A. 2014 11 25;111(47):16854–9. doi: 10.1073/pnas.1415396111 25385602 10.1073/pnas.1415396111 PMC4250133

[41] Acevedo A, Andino R. Library preparation for highly accurate population sequencing of RNA viruses. Nat Protoc. 2014 7;9(7):1760–9. doi: 10.1038/nprot.2014.118 24967624 10.1038/nprot.2014.118 PMC4418788

[42] Acevedo A, Brodsky L, Andino R. Mutational and fitness landscapes of an RNA virus revealed through population sequencing. Nature. 2014 1 30;505(7485):686–90. doi: 10.1038/nature12861 24284629 10.1038/nature12861 PMC4111796

[43] Yewdell JW. Monoclonal antibodies specific for discontinuous epitopes direct refolding of influenza A virus hemagglutinin. Mol Immunol. 2010 2;47(5):1132–6. doi: 10.1016/j.molimm.2009.10.023 20047763 10.1016/j.molimm.2009.10.023 PMC2814887

[44] Fazekas De St Groth S, White DO. An improved assay for the infectivity of in influenza viruses. J Hyg (Lond). 1958 3;56(1):151–62. 13525723 10.1017/s0022172400037621 PMC2217944

[45] Masella AP, Bartram AK, Truszkowski JM, Brown DG, Neufeld JD. PANDAseq: paired-end assembler for illumina sequences. BMC Bioinformatics. 2012 2 14;13:31 doi: 10.1186/1471-2105-13-31 22333067 10.1186/1471-2105-13-31 PMC3471323

[46] Kong Y. Btrim: a fast, lightweight adapter and quality trimming program for next-generation sequencing technologies. Genomics. 2011 8;98(2):152–3. doi: 10.1016/j.ygeno.2011.05.009 21651976 10.1016/j.ygeno.2011.05.009

[47] Li H, Durbin R. Fast and accurate short read alignment with Burrows-Wheeler transform. Bioinforma Oxf Engl. 2009 7 15;25(14):1754–60. 10.1093/bioinformatics/btp324 PMC2705234 19451168

[48] Quinlan AR, Hall IM. BEDTools: a flexible suite of utilities for comparing genomic features. Bioinforma Oxf Engl. 2010 3 15;26(6):841–2. 10.1093/bioinformatics/btq033 PMC2832824 20110278

[49] Zhou B, Donnelly ME, Scholes DT, St George K, Hatta M, Kawaoka Y, et al Single-reaction genomic amplification accelerates sequencing and vaccine production for classical and Swine origin human influenza a viruses. J Virol. 2009 10;83(19):10309–13. doi: 10.1128/JVI.01109-09 19605485 10.1128/JVI.01109-09 PMC2748056

