# Correction: Influenza A virus hemagglutinin glycosylation compensates for antibody escape fitness costs

**DOI:** 10.1371/journal.ppat.1007141

**Published:** 2018-06-20

**Authors:** Ivan Kosik, William L. Ince, Lauren E. Gentles, Andrew J. Oler, Martina Kosikova, Matthew Angel, Javier G. Magadán, Hang Xie, Christopher B. Brooke, Jonathan W. Yewdell

S2 Table is a duplicate of S1 Table. Please view the correct S2 Table here.

## Supporting information

S2 Table Minor allele frequency background level in primerID and Nextera/ViVan sequencing. (DOCX)
