# Supplementary figures and images for: Influenza A virus hemagglutinin glycosylation compensates for antibody escape fitness costs
Source: PLoS Pathog. 2018 Jan 18;14(1):e1006796. doi: 10.1371/journal.ppat.1006796 (PMC5773227; doi:10.1371/journal.ppat.1006796)

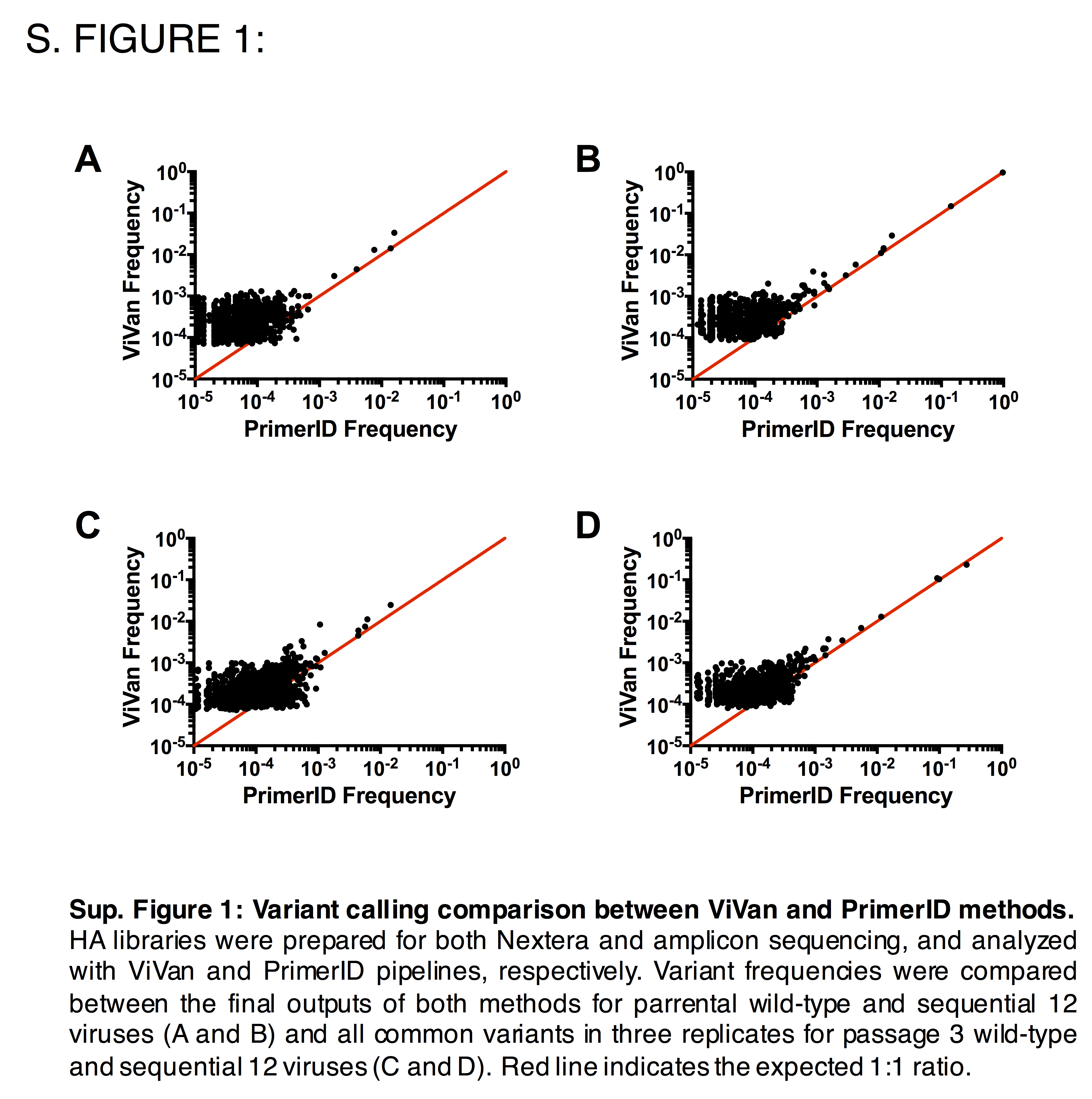

Supplement: S1 Fig — HA libraries were prepared for both Nextera and amplicon sequencing, and analyzed with ViVan and PrimerID pipelines, respectively. Variant frequencies were compared between the final outputs of both methods for parental wild-type and sequential 12 viruses (A and B) and all common variants in three replicates for passage 3 wild-type and sequential 12 viruses (C and D). Red line indicates the expected 1:1 ratio. (TIFF) [file ppat.1006796.s001.tiff]

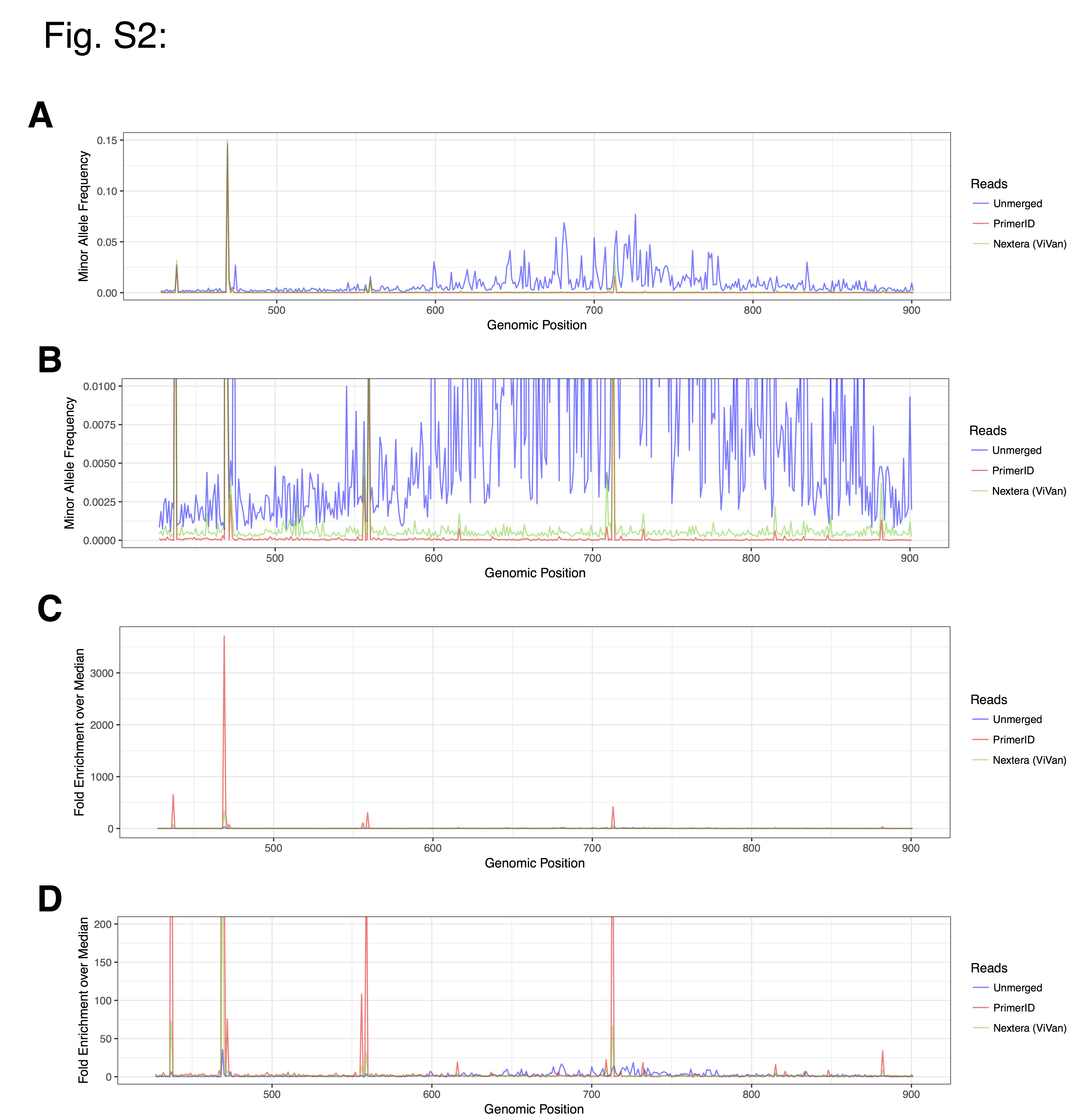

Supplement: S2 Fig — (A, B) Combined minor amino acid variant frequency is shown for each position of HA amplicon 2 region in SV12 parental sample for unmerged reads (before applying PrimerID), PrimerID consensus reads, and Nextera shotgun sequencing, evaluated by ViVan. (B) is a zoomed-in view of (A). (C, D) Fold enrichment of variant frequency over median variant frequency for the amplicon region, shown for the same three methods in (A) and (B). (D) is a zoomed-in view of (C). (TIF) [file ppat.1006796.s002.tif]

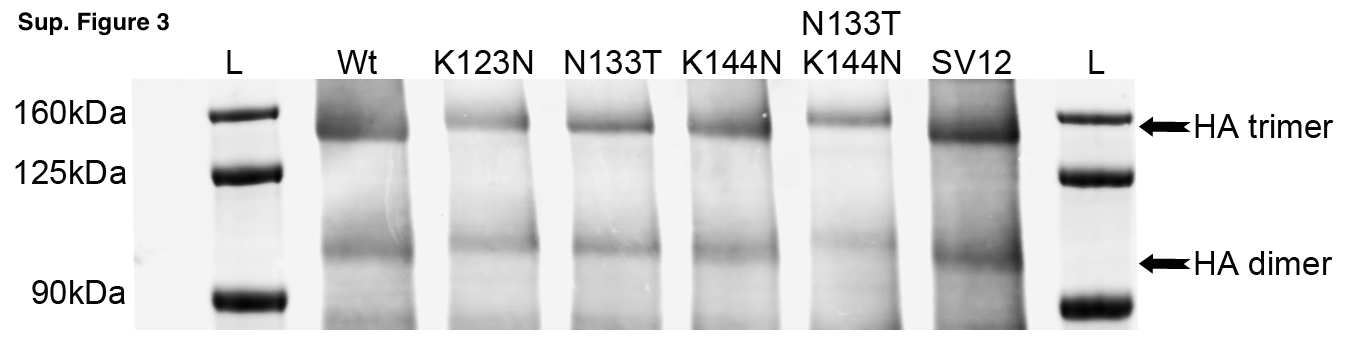

Supplement: S3 Fig — Immunoblot of purified virions as indicated electrophoresed under non-reducing conditions using the HA2 specific mAb RA5-22. (TIFF) [file ppat.1006796.s003.tiff]

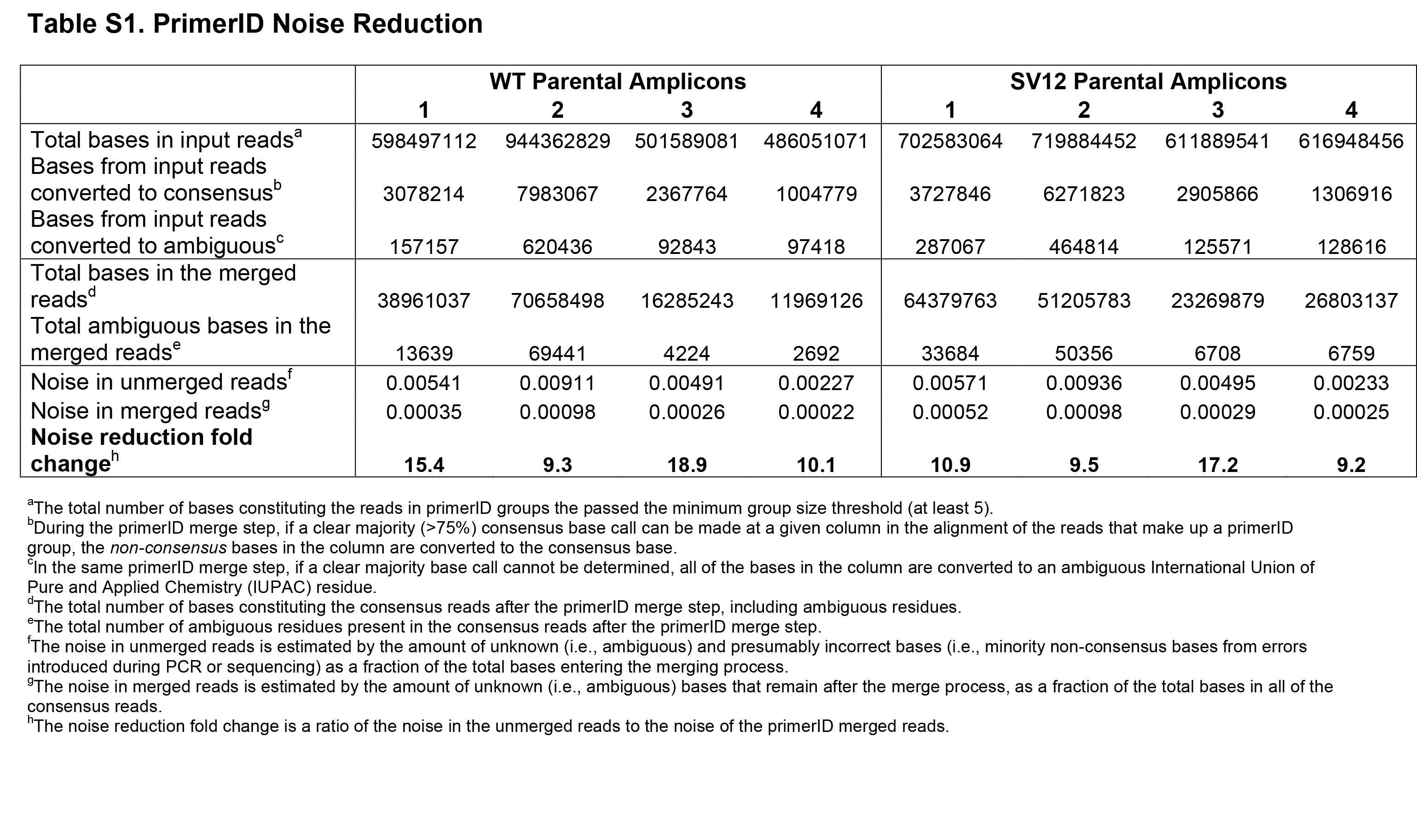

Supplement: S1 Table — (TIF) [file ppat.1006796.s004.tif]

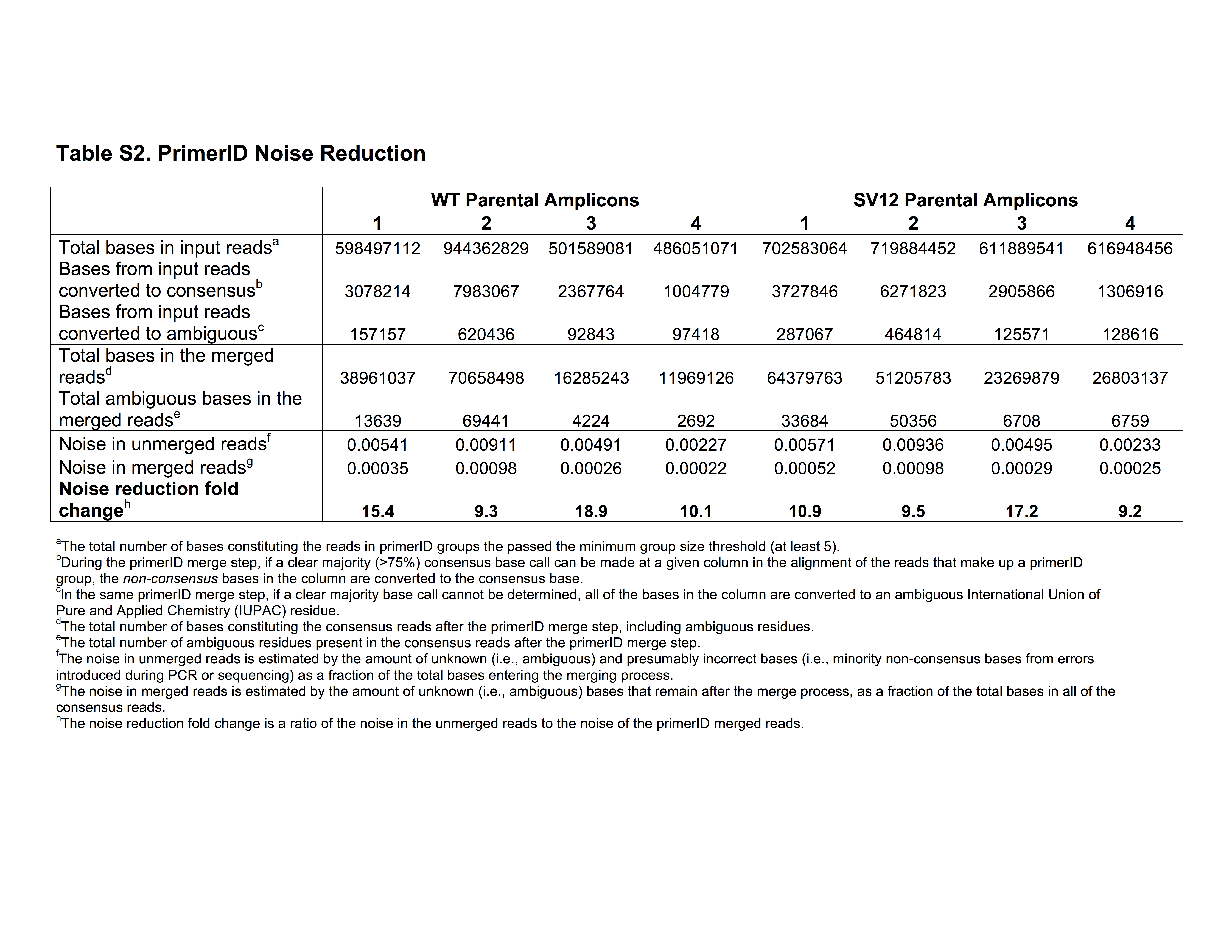

Supplement: S2 Table — (TIFF) [file ppat.1006796.s005.tiff]

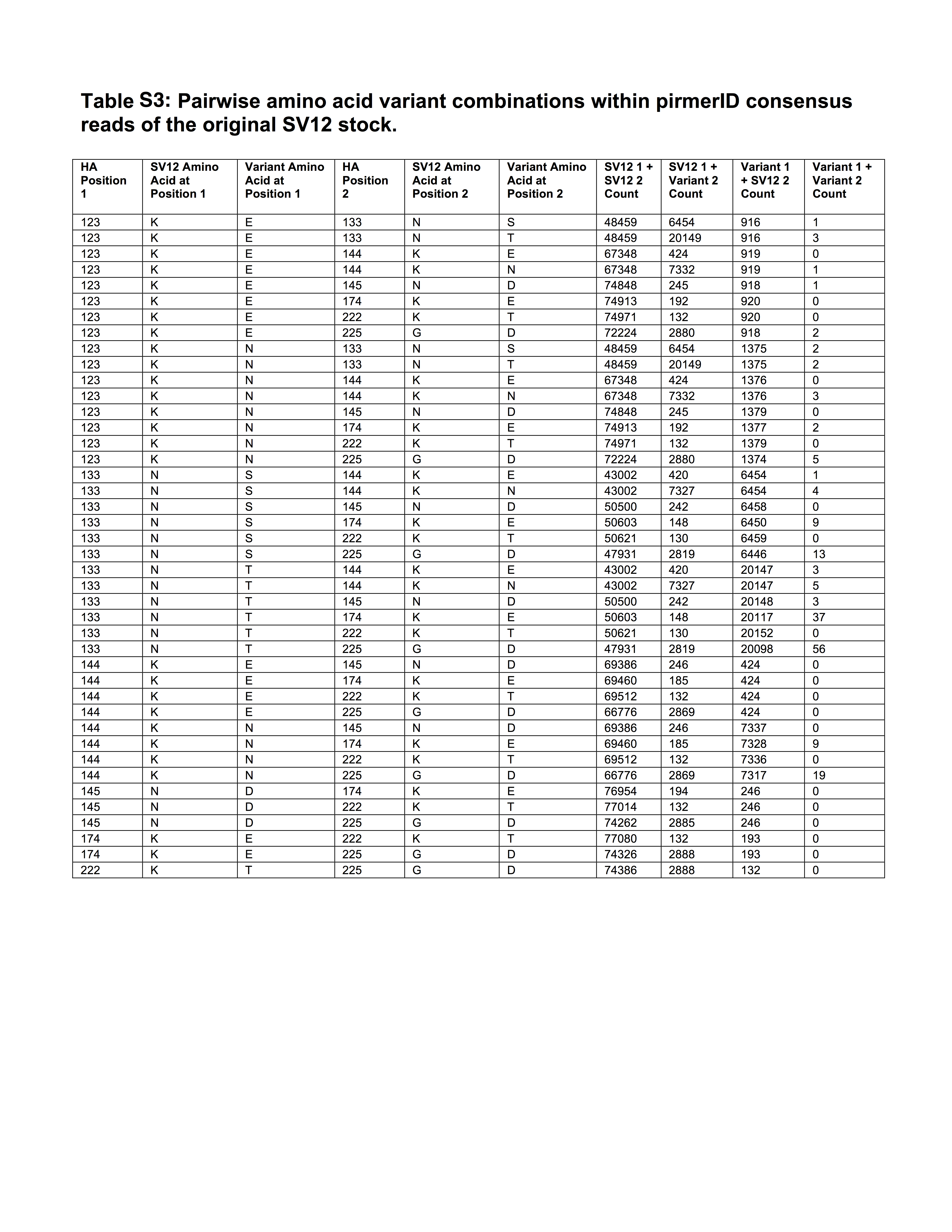

Supplement: S3 Table — Variant amino acids were evaluated pairwise to determine the frequency at which any two substitutions were observed in the same primerID consensus read. The combined counts across the two biological replicates that were sequenced are listed for each pairwise amino acid variant combination. (TIFF) [file ppat.1006796.s006.tiff]

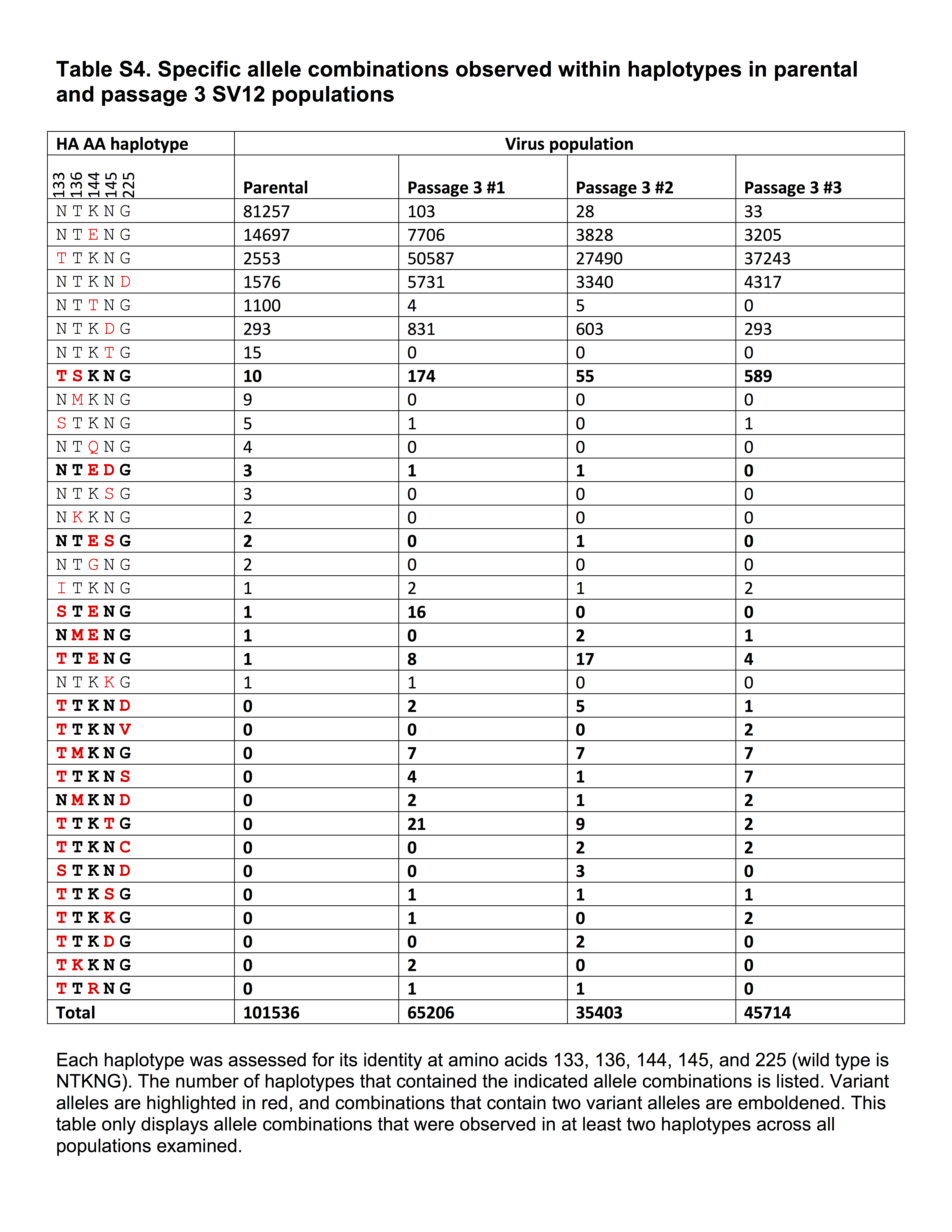

Supplement: S4 Table — Each primerID consensus read was assessed for its inferred amino acid identity at positions 133, 136, 144, 145, and 225 (wild type at each position is NTKNG, respectively). The number of primerID consesus reads that contained the indicated amino acid identities at these positions is listed. Variant amino acids are highlighted in red, and combinations that contain two variant amino acids are in bold face. This table only displays amino acid variant combinations that were observed in at least two primerID concensus reads across all populations examined. (TIFF) [file ppat.1006796.s007.tiff]

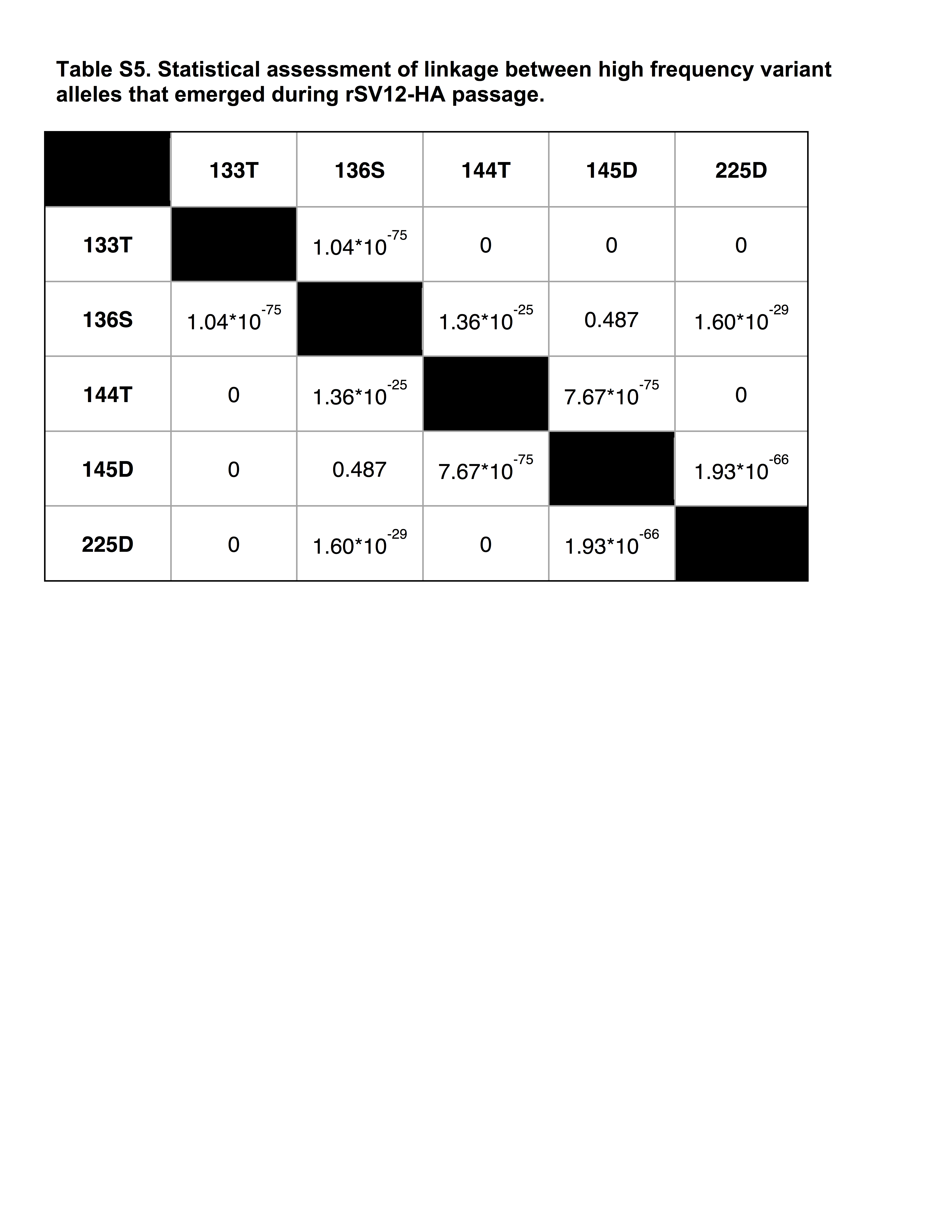

Supplement: S5 Table — Combined p-values from three replicate populations as determined by Fisher’s method. (TIFF) [file ppat.1006796.s008.tiff]
