# Supplementary material for: Correction: Influenza A virus hemagglutinin glycosylation compensates for antibody escape fitness costs
Source: PLoS Pathog. 2018 Jun 20;14(6):e1007141. doi: 10.1371/journal.ppat.1007141 (PMC6010253; doi:10.1371/journal.ppat.1007141)
Supplement: S2 Table — (DOCX) [file ppat.1007141.s001.docx]

**S2 Table. Minor Allele Frequency Background Level in PrimerID and Nextera/ViVan Sequencing**

|  | **SV12 Parental Amplicons** | | | | **WT Parental Amplicons** | | | |
| --- | --- | --- | --- | --- | --- | --- | --- | --- |
|  | **1** | **2** | **3** | **4** | **1** | **2** | **3** | **4** |
| Unmerged reads median MAF | 0.003075 | 0.004142 | 0.002919 | 0.002056 | 0.003132 | 0.004002 | 0.002872 | 0.002066 |
| PrimerID merged reads median MAF | 0.000034 | 0.000039 | 0.000057 | 0.000102 | 0.000045 | 0.000041 | 0.000082 | 0.000098 |
| Nextera ViVan median MAF | 0.000416 | 0.000437 | 0.000431 | 0.000456 | 0.000450 | 0.000465 | 0.000460 | 0.000478 |
| **Fold background difference in PrimerID vs Unmerged reads**^a^ | **89.9** | **107.5** | **50.8** | **20.1** | **69.4** | **97.8** | **34.9** | **21.0** |
| **Fold background difference in PrimerID vs Nextera ViVan**^b^ | **12.2** | **11.3** | **7.5** | **4.5** | **10.0** | **11.4** | **5.6** | **4.9** |
| PrimerID 95% confidence threshold^c^ | 0.000080 | 0.000099 | 0.000168 | 0.000211 | 0.000116 | 0.000089 | 0.000240 | 0.000287 |
| Nextera ViVan 95% confidence threshold^c^ | 0.000543 | 0.000535 | 0.000537 | 0.000594 | 0.000555 | 0.000549 | 0.000548 | 0.000586 |
| **Fold difference of confidence threshold for PrimerID vs Nextera ViVan**^d^ | **6.8** | **5.4** | **3.2** | **2.8** | **4.8** | **6.2** | **2.3** | **2.0** |
| Variants passing threshold in PrimerID^e^ | 31 | 26 | 11 | 16 | 4 | 8 | 4 | 3 |
| Variants passing threshold in Nextera ViVan^e^ | 94 | 107 | 77 | 64 | 97 | 111 | 107 | 82 |

^a^Fold difference was calculated by taking the ratio of the median PrimerID combined minor allele frequency (cMAF) within an amplicon to the median MAF calculated from unmerged amplicon reads. Reads belonging to primerID groups that fall below the minimum group size threshold were not included.

^b^Fold difference was calculated by taking the ratio of the median PrimerID cMAF within an amplicon to the median combined MAF from Nextera ViVan analysis for the same region.

^c^The 95% confidence threshold was computed by taking the median value for each amplicon region of the upper bracket of the 95% confidence interval for the cMAF at each position, computed from the binomial model in R.

^d^The difference was calculated by taking the ratio of the confidence threshold for Nextera/ViVan to the threshold for PrimerID.

^e^The number of variants passing the threshold was determined by filtering for variants where the lower bracket of the 95% confidence interval for the cMAF at a particular position is higher than the upper bracket of the 95% confidence threshold of the median cMAF.
